# Mitochondrial Iron Transporters (MIT1 and MIT2) Are Essential for Iron Homeostasis and Embryogenesis in *Arabidopsis thaliana*

**DOI:** 10.3389/fpls.2019.01449

**Published:** 2019-11-25

**Authors:** Anshika Jain, Zachary S. Dashner, Erin L. Connolly

**Affiliations:** ^1^Department of Biological Sciences, University of South Carolina, Columbia, SC, United States; ^2^Department of Plant Science, The Pennsylvania State University, University Park, PA, United States

**Keywords:** mitochondria, iron, mitochondrial iron transport, arabidopsis, iron homeostasis

## Abstract

Iron (Fe) is an essential nutrient for virtually all organisms, where it functions in critical electron transfer processes, like those involved in respiration. Photosynthetic organisms have special requirements for Fe due to its importance in photosynthesis. While the importance of Fe for mitochondria- and chloroplast-localized processes is clear, our understanding of the molecular mechanisms that underlie the trafficking of Fe to these compartments is not complete. Here, we describe the Arabidopsis mitochondrial iron transporters, MIT1 and MIT2, that belong to the mitochondrial carrier family (MCF) of transport proteins. MIT1 and MIT2 display considerable homology with known mitochondrial Fe transporters of other organisms. Expression of MIT1 or MIT2 rescues the phenotype of the yeast *mrs3mrs4* mutant, which is defective in mitochondrial iron transport. Although the Arabidopsis *mit1* and *mit2* single mutants do not show any significant visible phenotypes, the double mutant *mit1mit2* displays embryo lethality. Analysis of a *mit1* ^−−^ */mit2* *^+^* ^−^ line revealed that MIT1 and MIT2 are essential for iron acquisition by mitochondria and proper mitochondrial function. In addition, loss of *MIT* function results in mislocalization of Fe, which in turn causes upregulation of the root high affinity Fe uptake pathway. Thus, MIT1 and MIT2 are required for the maintenance of both mitochondrial and whole plant Fe homeostasis, which, in turn, is important for the proper growth and development of the plant.

## Introduction

Iron (Fe) is an essential element that is required for numerous biochemical processes in cells. It readily accepts and donates electrons, and functions as a part of redox centers where it serves as a cofactor for various enzymes and proteins. The molecular details of Fe uptake in plants are reasonably well described (Kobayashi and Nishizawa 2012; Jeong et al., 2017). Grasses like rice, barley, and maize utilize a chelation-based strategy in which Fe uptake from the rhizosphere is mediated by phytosiderophores such as deoxymugineic acid. Studies in rice and barley have shown that in response to iron limitation, phytosiderophores (PSs) are exported by transporter of mugineic acid (TOM1) and Fe-PS complexes are subsequently imported by YS1, a member of the oligopeptide transporter family (Curie et al., 2001; Inoue et al., 2009; Nozoye et al., 2011; Connorton et al., 2017). Dicots such as Arabidopsis, on the other hand, use a reduction-based strategy in which insoluble ferric chelates in the rhizosphere are solubilized *via* proton extrusion mediated by the H^+^ ATPase AHA2 and coumarin secretion (Santi and Schmidt, 2009; Jeong et al., 2017). Upon solubilization, the rhizospheric ferric chelates are reduced to ferrous ions by a root surface-localized ferric chelate reductase, FRO2 (Robinson et al., 1999; Connolly et al., 2003) and subsequently imported into the root epidermal cells by a high affinity iron transporter, IRT1 (Eide et al., 1996; Vert et al., 2002; Jeong and Connolly, 2009). In addition to its role in iron uptake from the soil, IRT1 also functions in the import of other divalent cations such as Mn^2+^, Zn^2+^, Co^2+^ and Cd^2+^ (Eide et al., 1996; Korshunova et al., 1999; Vert et al., 2002; Grossoehme et al., 2006).

Cells prioritize delivery of Fe to mitochondria to maintain the proper functioning of iron-requiring biochemical processes such as Fe-S cluster biosynthesis, apoptosis, and respiration. Numerous respiratory complex subunits utilize iron (Fe-S clusters, heme, and/or non-heme iron) as their cofactors (Philpott and Ryu, 2014). Mitochondria also function as manufacturing sites for synthesis of Fe-S clusters in all organisms and heme groups in non-photosynthetic organisms (Yoon and Cowan, 2004; Balk and Pilon, 2011; Rouault, 2014; Rouault, 2015). Despite its importance, the mechanisms that control mitochondrial Fe metabolism are not fully understood in plants. While Fe export out of the mitochondria is an open question in all living systems, several reports have shed light on iron import to mitochondria (Froschauer et al., 2013; Jain and Connolly, 2013; Vigani et al., 2019).

The yeast mitoferrin proteins MRS3 and MRS4 (Mitochondrial RNA Splicing proteins) were the first proteins shown to function in mitochondrial iron uptake (Foury and Roganti, 2002). These high affinity iron transporters move iron across the mitochondrial inner membrane in a pH- and concentration-dependent manner (Froschauer et al., 2009). The accumulation of Fe, heme, and Fe-S clusters in yeast mitochondria was shown to be directly proportional to the expression levels of MRS3 and MRS4 in yeast overexpression lines (Foury and Roganti, 2002; Muhlenhoff et al., 2003). MRS3 and MRS4 deletion mutants display a growth phenotype only under Fe-limiting conditions (Muhlenhoff et al., 2003), suggesting the presence of a low affinity Fe transporter that functions under Fe-sufficient conditions. Rim2, a pyrimidine nucleotide exchanger, has been shown to function as an alternative route for mitochondrial Fe transport in yeast (Yoon et al., 2011). Additionally, a recent study utilized *mrs3mrs4* mutant cells to identify a low molecular mass pool of Fe that serves as the feedstock for Fe-S cluster assembly and heme synthesis in yeast (Moore et al., 2018). This study also showed that Fe homeostasis is disrupted in *mrs3mrs4* mutants (Moore et al., 2018).

Homologs of yeast mitoferrins have been described in zebrafish (Shaw et al., 2006; Paradkar et al., 2009), Drosophila (Metzendorf et al., 2009) and rice (Bashir et al., 2011). Drosophila mitoferrin (Dmfrn) is essential for spermatogenesis and loss of Dmfrn leads to male sterility (Metzendorf et al., 2009). The zebrafish *frascatii* (*mfrn1*) mutant displays impaired heme synthesis which leads to severe defects in erythropoiesis and subsequent death of the embryo (Shaw et al., 2006). A related protein, Mfrn2, fails to rescue the defective erythropoetic phenotype of the *mfrn1* mutant. While the role of Mfrn2 is not clear at the organismal level, both Mfrn1 and Mfrn2 were shown to be responsible for Fe transport in the mitochondria of nonerythroid cells (Paradkar et al., 2009). A recent *in vitro* study revealed that Mfrn1 transports free iron (rather than chelated iron complexes) in addition to Co, Cu, Zn, and Mn (Christenson et al., 2018).

The mitoferrin ortholog identified in rice (mitochondrial iron transporter [MIT]) was shown to be responsible for mitochondrial iron acquisition (Bashir et al., 2011). While *mit* knockout mutants are embryo lethal, *mit* knock-down lines exhibit a poor growth phenotype, reduced mitochondrial iron, and increased total Fe in the shoots, suggesting that Fe is mislocalized in *mit* cells. The expression level of the gene coding for the Vacuolar Iron Transporter1 (*VIT1*) is upregulated in the absence of *MIT* in rice (Bashir et al., 2011). Overall, the loss of mitoferrin function in different species results in severely retarded growth and embryo lethal phenotypes (Muhlenhoff et al., 2003; Shaw et al., 2006; Paradkar et al., 2009; Bashir et al., 2011).

In this study, we report the characterization of previously unidentified mitochondrial iron transporters in dicots for the first time. We cloned and characterized two mitochondrial iron transporters (MIT1 and MIT2) from Arabidopsis and demonstrate their importance for mitochondrial iron acquisition as well as for the maintenance of Fe homeostasis in plants. We observed that the loss of MIT1 and MIT2 together results in reduced levels of mitochondrial iron, as well as impaired mitochondrial biochemical function as evidenced by changes in the relative abundance of various respiratory subunits and aconitase levels. Our results also show that MIT1 and MIT2 function redundantly and are essential for embryogenesis. Although rice and Arabidopsis MITs show functional similarities, here we describe key differences between mitochondrial iron transport in dicots and monocots.

## Materials and Methods

### Plant Lines and Plant Growth Conditions

T-DNA insertion mutants [SALK_013388 for *MIT1*(At2g30160) and SALK_096697 for *MIT2* (At1g07030)] were obtained from the Arabidopsis Biological Research Center (ABRC). The *mit1mit2* double mutant was generated using artificial microRNA technology as described below (Schwab et al., 2006). Wild type Arabidopsis (Col-*0* or Col *gl-1*) was used as a control in all the experiments. Seeds were surface sterilized with 25% bleach and 0.02% SDS. After thorough washing with dH2O, seeds were imbibed in the dark for 2 days at 4°C. Seedlings were grown on Gamborg’s B5 media (Phytotechnology Laboratories) supplemented with 2% sucrose, 1mM MES, and 0.6% agar, pH 5.8 for 2 weeks under constant light (80 mmol/m^−2^/s^−2^) at 22°C. To induce iron deficiency, plants were transferred after 2 weeks from B5 media to 300 µM ferrozine [3-(2-pyridyl)-5,6-diphenyl-1,2,4 triazine sulfonate] containing media (Fe-deficient media) for an additional 3 days as described previously (Connolly et al., 2002). Plants were also grown with and without added Fe (but without an Fe chelator) as follows: 1/2X MS media, 0.6% agar, pH 5.8 supplemented with either 50 µM Fe(III)-EDTA (Fe-sufficient media) or no additional Fe (Fe drop-out media) for 2.5 weeks.

Plants were grown in Metro-Mix360/perlite/vermiculite (5:1:1) under 16 hr light; 8hr dark or were grown hydroponically under constant light. The composition of the hydroponics nutrient solution was as follows: 0.75 mM K_2_SO_4_, 0.1 mM KH_2_PO_4_, 2.0 mM Ca(NO_3_)_2_, 0.65 mM MgSO_4_, 0.05 mM KCl, 10 µM H_3_BO_3_, 1 µM MnSO_4_, 0.05 µM ZnSO_4_, 0.05 µM CuSO_4_, 0.005 µM (NH_4_)_6_Mo_7_O_24_, with no added Fe (Fe drop-out media) (Kerkeb et al., 2008); media was replaced weekly.

### Cloning

For the generation of *35S-MIT1-YFP* and *35S-MIT2-YFP* constructs, the full length cDNAs for *MIT1* and *MIT2*, already cloned in an entry vector (D-TOPO, Life Technologies) were ordered from The Arabidopsis Information Resource (TAIR). In order to obtain *MIT-YFP* fusion constructs, full length CDS lacking the stop codons of *MIT1* and *MIT2* were amplified using gene specific primers (*MIT1* FP: 5′ CACCATGGCAACAGAAGCAACAACC-3′, *MIT1* RP: 5′AGCTGCGTTTGCTTCACCATTGAG-3′, *MIT2* FP: 5′-CACCATGGCTACGGAGGCTACAAC-3′, *MIT2* RP: 5′-GGCAG​AGTTTGAATCGACATTGAAG-3′). These DNA fragments were then subcloned into pENTER/D-TOPO and recombined into pEARLY Gateway101 (Life Technologies); these constructs were then transformed into *Agrobacterium tumefaciencs GV3101* using standard cloning and transformation techniques (Koncz and Schell, 1986).

To generate the *pMIT1-GUS* and *pMIT2-GUS* constructs, primers were designed to amplify a 1.5-kb region upstream of *MIT1* and a 0.75-kb region (limited by the presence of another gene upstream on the chromosome) upstream of *MIT2* from the genomic DNA (*pMIT1* FP: 5′ GGTACCCTTTAGTTTAACCGCCGCAT-3′, *pMIT1* RP: 5′GAATTCTTTCTCTATCAATGCAAACCAGAA-3′, *pMIT2* FP: 5′GGTACCTTGTGGAAGAAAGATCAAATCTTG-3′, *pMIT2* RP: 5′GAATTCATCATCAACACAAACCTGGAAA-3′). *pMIT1* was cloned into HindIII/EcoRI sites and similarly *pMIT2* was cloned into HindIII/BamH1 sites of pCAMBIA1381Xa. These clones were transformed into Agrobacterium *GV3101*. Arabidopsis (Col *gl*-*1*) was then transformed using the floral dip protocol (Clough and Bent, 1998).

Artificial microRNA lines designed to knockdown expression of both *MIT1* and *MIT2* (*amiRmit1mit2*) were developed using the Web MicroRNA Designer (Schwab et al., 2006) as previously reported (Bernal et al., 2012). The targeting microRNA (TATATAGTAGCGAAAACGCCG) was designed to target both *MIT1* and *MIT2* using the following primers: *mit1mit2miR*-sense: 5′GATATATAGTAGCGAAAACGCCGTCTCTCTTTTGTATTCC3′, *mit1mit2miR*-antisense: 5′GACGGCGTTTTCGCTACTATATATCAAAGAGAATCAATGA3′ and mit1mit2miR*sense: 5′GACGACGTTTTCGCTTCTATATTTCACAGGTCGTGATATG3′, and mit1mit2miR*antisense: 5′GAAATATAGAAGCGAAAACGTCGTCTACATATATATTCCT3′. The fragment was amplified by overlapping PCR using a template plasmid (pRS300), a kind gift from Dr. Detlef Weigel [http://www.weigelworld.org; (Schwab et al., 2006)]. The amplicon was further cloned into the Not1 and Xho1 sites of 35S-pBARN (LeClere and Bartel, 2001).

For the yeast complementation assay, the *MIT1* and *MIT2* cDNAs were amplified from Col*-0* cDNA and cloned into the BamHI and XhoI sites of pRS426 (a kind gift from Dr. Jerry Kaplan) using the following sets of primers. *MIT1* FP: 5′-CGCGGATCCATGGTAGAAAACTCGTCGAGTAATAATTCAACAAGGCCAATTCCAGCAATACCTATGGATCTACCCTTTCATCCAGCAATCATCGTT-3′,*MIT1* RP: 5′-CCGCTCGAGTTATCACTTGTCATCGTCATCCTTGTAATCACCACCAGCTGCGTTTGCTTCACCATT-3′, *MIT2* FP: 5′-CGCGGATCCATGGTAGAAAACTCGTCGAGTAATAATTCAACAAGGCCAATTCCAGCAATACCTATGGATCTACCCCCGGATTTCAAACCGGAAATC-3′, *MIT2* RP: 5′-CCGCTCGAGTTATCACTTGTCATCGTCATCCTTGTAATCACCACCGGCAGTGTTTGAATCGACATT-3′.

### Subcellular Localization

Onion peel epidermis cells were transiently transformed with the *35S-MIT1-YFP* and *35S-MIT2-YFP* constructs as described (Sun et al., 2007). The transformed onion peels were rinsed with water and stained with 150 nM MitoTracker Orange (CMTMRos; Life Technologies). Fluorescence images were generated using a Zeiss LSM 700 meta confocal system. An argon laser at 488 nm and at 535 nm provided the excitation for YFP and MitoTracker Orange (CMTMRos), respectively. Emission of YFP was collected between 505 and 530 nm, and emission of MitoTracker was collected between 585 and 615 nm. Zen lite 2011 software was used to analyze the fluorescence images.

### GUS Histochemical Staining

Two-week-old seedlings of the homozygous T4 generation were used for GUS histochemical staining using X-Gluc (5-bromo-4-chloro-3-indonyl β-D glucoronide; Thermo Scientific) as a substrate as described in (Jefferson et al., 1987; Divol et al., 2013). The staining was performed on five independent, single-insertion, homozygous lines grown on B5 media, and representative lines are shown.

### RNA Isolation and Transcript Analysis

Total RNA was extracted from 100 mg frozen tissue of 2-week-old seedlings grown on standard Gamborg’s B5 media using TRIzol reagent (Sigma). DNase1 treatment (New England Biolabs) was conducted on 3.5 µg of the total RNA for 15 min. Superscript First strand Synthesis system (Life Technologies) was used to prepare the cDNA from total RNA according to the manufacturer’s instructions (Mukherjee et al., 2006). Quantitative real-time PCR (qRT-PCR) was performed as described (Fraga et al., 2008). The following primers were used for q-RT PCR of *MIT1* and *MIT2* on the T-DNA mutants and the *amiRmit1mit2* mutant *MIT1* FP: CATGCTGTTGGAGCAGAGGA, *MIT1* RP: CACAACCACACACACCCTGA; *MIT2* FP: AGTTGCAATGTCAGGGTGTGT, *MIT2* RP: CGGGAGCCATCCCCTTAGAA. Primers to study the levels of *MIT1* and *MIT2* in the roots and shoots of WT plants are as follows: *MIT1* FP: 5′-AGACGCAGTTGCAATGTCAG-3′, *MIT1* RP: 5′-AGCCATCCTCTAGCAAGTCCT-3′, *MIT2* FP: 5′-CGCTTGATGTTGTCAAGACG-3′, *MIT2* RP: 5′-AGGAGCATGGAAGAGCATTCT-3′, *Actin* FP: 5′-CCTTTGTTGCTGTTGACTACGA-3′, *Actin* RP: 5′-GAACAAGACTTCTGGGCATCT-3′. Semi-qRT-PCR was performed using the following primers (Marone et al., 2001). *MIT1*: 5′-CACCATGGCAACAGAAGCAACAACC-3′, *MIT1* RP: 5′-AGCTGCGTTTGCTTCACCATTGAG-3′, *MIT2* FP: 5′- ATGGCTACGGAGGCTACAAC-3′, *MIT2* RP: 5′-GGCAGAGTTTGAATCGACATTGAAG-3′.

### Yeast Complementation Assay

The following primers were designed to tag the protein with FLAG and substitute the 1st- 22 amino acids of MIT1 and MIT2 with the yeast MRS3 leader peptide to ensure proper targeting of the Arabidopsis proteins to the mitochondria of yeast cells (Shaw et al., 2006). *MIT1* FP: 5′ CGCGGATCCATGGTAGAAAACTCGTCGAGTAAT AATTCAACAAGGCCAATTCCAGCAATACCTATGGATCTACCCTTTCATCCAGCAATCATCGTT 3′, *MIT1* RP: 5′CCGCTCGAGTTATCACTTGTCATCGTCATCCTTGTAATCACCACCAGC TGCGTTTGCTTCACCATT 3′; 5′ *MIT2* FP: CGCGGATCCATGGTAGAAAACTCGTCGAGT AATAATTCAACAAGGCCAATTCCAGCAATACCTATGGATCTACCCCCGGATTTCAAACCGGAAATC 3′, *MIT2* RP: 5′ CCGCTCGAGTTATCACTTGTCATCGTCATCCTTGTAATCACCACCGGCAGTGTTTGAATCGACATT 3′. The *mrs3mrs4* yeast strain was transformed with the empty vector (pRS426-ADH), the vector containing the *MRS3* ORF (positive control) or the vector containing *MIT1-FLAG* or *MIT2-FLAG* (Pan et al., 2004). Yeast strains were grown in liquid SD-URA medium, and serial dilutions were prepared (OD_600_ 1, 0.1 and 0.01). 10µl of the dilutions were plated as spots on the SD-URA plates containing either 0.1mM FeSO_4_ or 0.1mM bathophenanthroline disulfonate (BPS) as described (Li and Kaplan, 2004). The plates were incubated at 30°C for 4 days.

### Ferric Reductase Assay

For the ferric reductase activity assay, plants were grown on Gamborg’s B5 media for 2 weeks and then transferred to Fe-sufficient (50 µM Fe-EDTA) or Fe-deficient media (300 µM ferrozine) for 3 days (Connolly et al., 2003). For the measurement of reductase activity, the roots of the intact seedlings were submerged in 300µl assay solution comprised of 50 µM Fe(III) EDTA and 300µM ferrozine and placed in the dark for 20 min. The absorbance of the assay solution was then measured at 562 nm, and the activity was normalized to the fresh weight of the roots (Robinson et al., 1999; Connolly et al., 2003). Reported data are based on 10 biological replicates. A Student’s *t*-test was used to perform the statistical analysis.

### Mitochondrial Fractionation and Purification

Mitochondria were prepared from seedlings grown in B5 or 1/2X MS media (with or without Fe) for 2.5 weeks. A total of 40 to 50 g of tissue was ground in 100 ml of ice-cold extraction buffer containing 0.3 M sucrose, 25 mM MOPS pH 7.5, 0.2% (w/v) BSA, 0.6% (w/v) polyvinyl-pyrrolidone 40, 2 mM EGTA, and 4 mM cysteine. All procedures were carried out at 4°C. The extract was filtered through two layers of Miracloth and centrifuged at 6,500*g* for 5 min. The supernatant was then further centrifuged at 18,000*g* for 15 min. The pellet, thus obtained was gently resuspended in extraction buffer, and the aforementioned centrifugation steps were repeated again. The resulting crude organelle pellet was resuspended in the extraction buffer and layered on a 32% (v/v) continuous Percoll gradient solution (0.25M sucrose, 10 mM MOPS, 1 mM EDTA, 0.5% PVP-40, 0.1% BSA, 1 mM glycine). The gradient was centrifuged at 40,000*g* for 2 h 30 min and the mitochondria, visible as a whitish/light-brown ring, were collected. The purified mitochondria were washed twice by resuspending in wash buffer containing 0.3 M sucrose, 5 mM MOPS followed by centrifugation at 18,000*g* for 15 min. The final mitochondrial pellet was resuspended in wash buffer containing 100 mM PMSF (Branco-Price et al., 2005). Mitochondrial protein concentrations were determined using the BCA assay (Pierce).

### Elemental Analysis

For elemental analysis of mitochondria, samples were digested overnight in 250 µl concentrated HNO_3_ and 50 µl concentrated HCl at 60°C. The digested samples were centrifuged at 14,000*g* for 1 min, and the supernatant was diluted to obtain a final concentration of 2.5% HNO_3_. Samples were analyzed on a Thermo Elemental PQ ExCell ICP-MS using a glass conical nebulizer drawing 1 ml per min. The activity of three biological replicates was averaged for each genotype. The elemental analysis on the 44-day-old aerial tissues of soil-grown plants was conducted using ICP-MS at the University of Aberdeen as described (Lahner et al., 2003). A Student’s *t*-test was used to perform the statistical analysis.

### Western Blot Analysis

Protein lysates (25 µg) were separated by SDS-PAGE and transferred to PVDF (Fisher Scientific) membrane by electroblotting. Membranes were probed with IRT1 (Connolly et al., 2002), ferritin, actin, PSB-a, IDH, COX2, Histone3 (Agrisera), or aconitase antibodies and chemiluminescence detection was carried out as described (Connolly et al., 2002). Two micrograms of mitochondrial proteins were separated for aconitase antibody detection. Aconitase antibody was a kind gift from Dr. Janneke Balk (Luo et al., 2012).

### Blue Native Polyacrylamide Gel Electrophoresis (BN-PAGE) and In-Gel Assay

20 µg of the resuspended mitochondrial fraction enriched in respiratory complexes was subjected to BN-PAGE according to (Schagger and von Jagow, 1991) and the in-gel assay for complex I was carried out according to (Sabar et al., 2005). Gels were incubated with 0.2 mM NADH (Sigma) and 0.1% (w/v) nitroblue tetrazolium (Sigma) in 0.1 M Tris-HCl 7.4 for complex I/NADH dehydrogenase activities. The relative intensities of the complex I bands were calculated using ImageJ software (Schneider et al., 2012).

## Results

### MIT1 and MIT2 Functionally Complement a Yeast Mitoferrin Mutant

**Figure 1:**
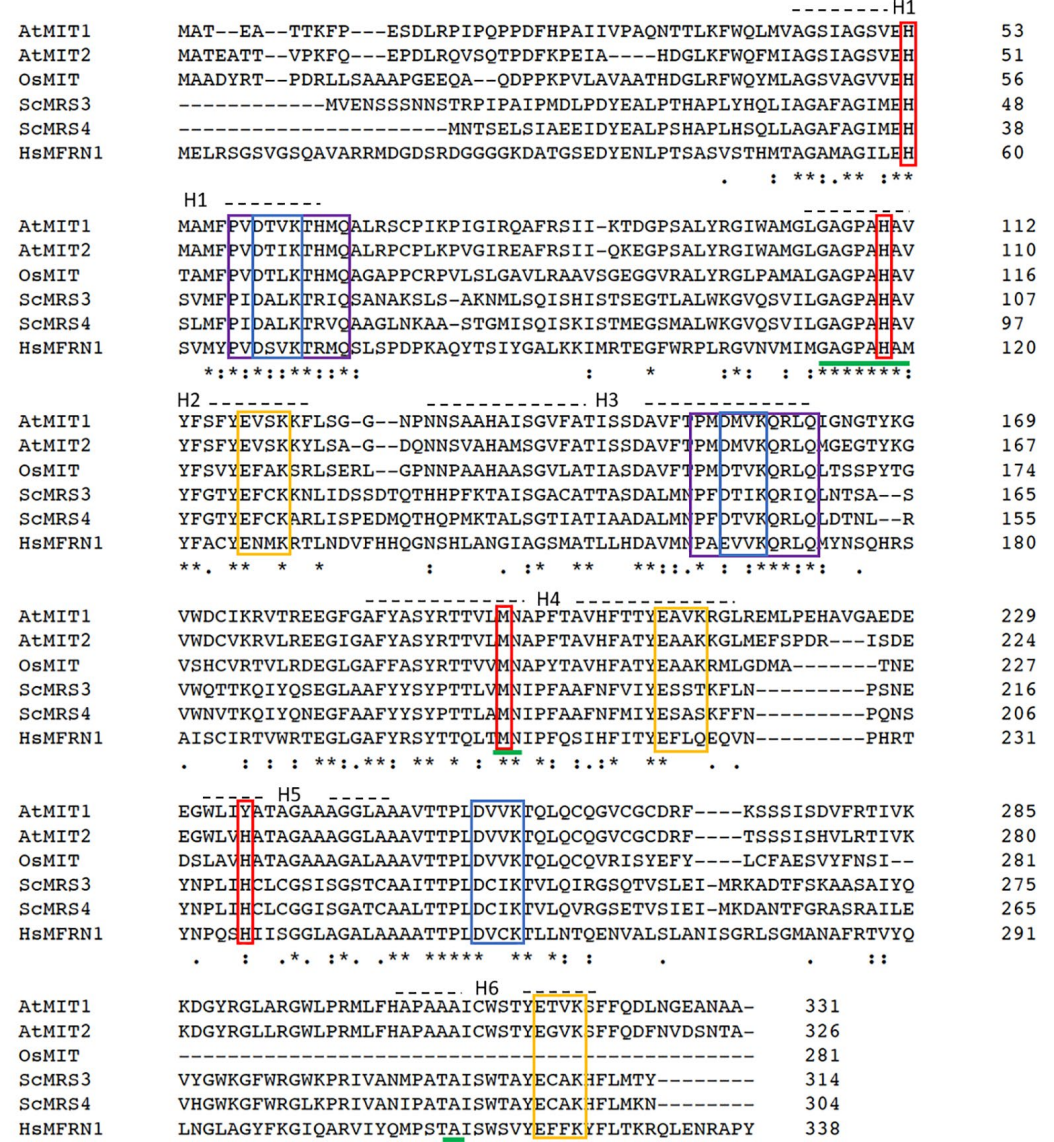
Mitochondrial iron transporters in Arabidopsis exhibit sequence conservation for the residues significant for mitochondrial Fe transport. Sequence alignment *of* Arabidopsis AtMIT1 and AtMIT2 with mitoferrins from rice (OsMIT), yeast (ScMRS3, ScMRS4) and human (HsMfrn1) generated using CLUSTAL O. Sequences of the six predicted transmembrane helices on these proteins are depicted by H1-H6. The Mitochondrial Energy Transfer Signature (METS-P-x-[DE]-x-[LIVAT]-[RK]-x-[LRH]-[LIVMFY]-[QGAIVM]) identified in AtMIT1 and AtMIT2 is indicated by the purple boxes. The putative Fe-binding motifs are underlined in green, and the residues implicated in Fe transport are indicated in red boxes (MIT1 Fe binding motifs: H53, H110, M196, Y235). Conserved [DE]-xx-[RK] motifs putatively forming the salt bridges on the cytosolic and the matrix side of the membrane are indicated in yellow and blue boxes, respectively. An * (asterisk) indicates positions which have a single, fully conserved residue. A : (colon) indicates conservation between groups of strongly similar properties (roughly equivalent to scoring > 0.5 in the Gonnet PAM 250 matrix). A . (period) indicates conservation between groups of weakly similar properties (roughly equivalent to scoring =< 0.5 and > 0 in the Gonnet PAM 250 matrix).

A sequence homology-based search for yeast mitoferrin (MRS3 and MRS4) orthologs in the *Arabidopsis* genome yielded two proteins—MIT1 (At2g30160) and MIT2 (At1g07030). MIT1 and MIT2 are 81% identical to each other at the amino acid level and share 38% sequence identity to yeast and 32% identity with zebrafish mitoferrins. These proteins belong to the mitochondrial substrate carrier family (MCF) of protein transporters and exhibit their characteristic Mitochondrial Energy Transfer Signature (METS-P-x-[DE]-x-[LIVAT]-[RK]-x-[LRH]-[LIVMFY]-[QGAIVM]) on the matrix side of the protein (**Figure 1**) (Nelson et al., 1998; Millar and Heazlewood, 2003). Additionally, MIT1 and MIT2 also exhibit the highly conserved putative signature Fe binding motifs (GXXXAHXXY, MN, and A) on transmembrane helices II, IV, and VI, respectively (Walker, 1992; Kunji and Robinson, 2006). Two mutagenesis-based studies have identified the residues important for Fe transport in yeast MRS3 and *O. niloticus* Mfrn1. The results from these studies report the importance of three conserved histidines (H48, H105, and to a lesser extent, H222 in MRS3) and a methionine (M207 in Mfrn1) in the transport of Fe (Brazzolotto et al., 2014; Christenson et al., 2018). The H222 in MRS3 is conserved in MIT2, however, is replaced by another potential Fe-ligand, a tyrosine, in MIT1. Furthermore, two motifs ([DE]-xx-[RK]) that likely form salt-bridge networks on each side of the membrane have been previously hypothesized to facilitate solute transport by MCF proteins. Mutagenesis of the residues building the putative salt-bridge networks were shown to either affect the integrity of the protein or result in loss of transport activity (Christenson et al., 2018). Interestingly, these residues were also identified in MIT1 and MIT2 (**Figure 1**). Thus, despite the relatively low sequence identity between the orthologs, the residues important for Fe transport appear to be conserved across species.

**Figure 2:**
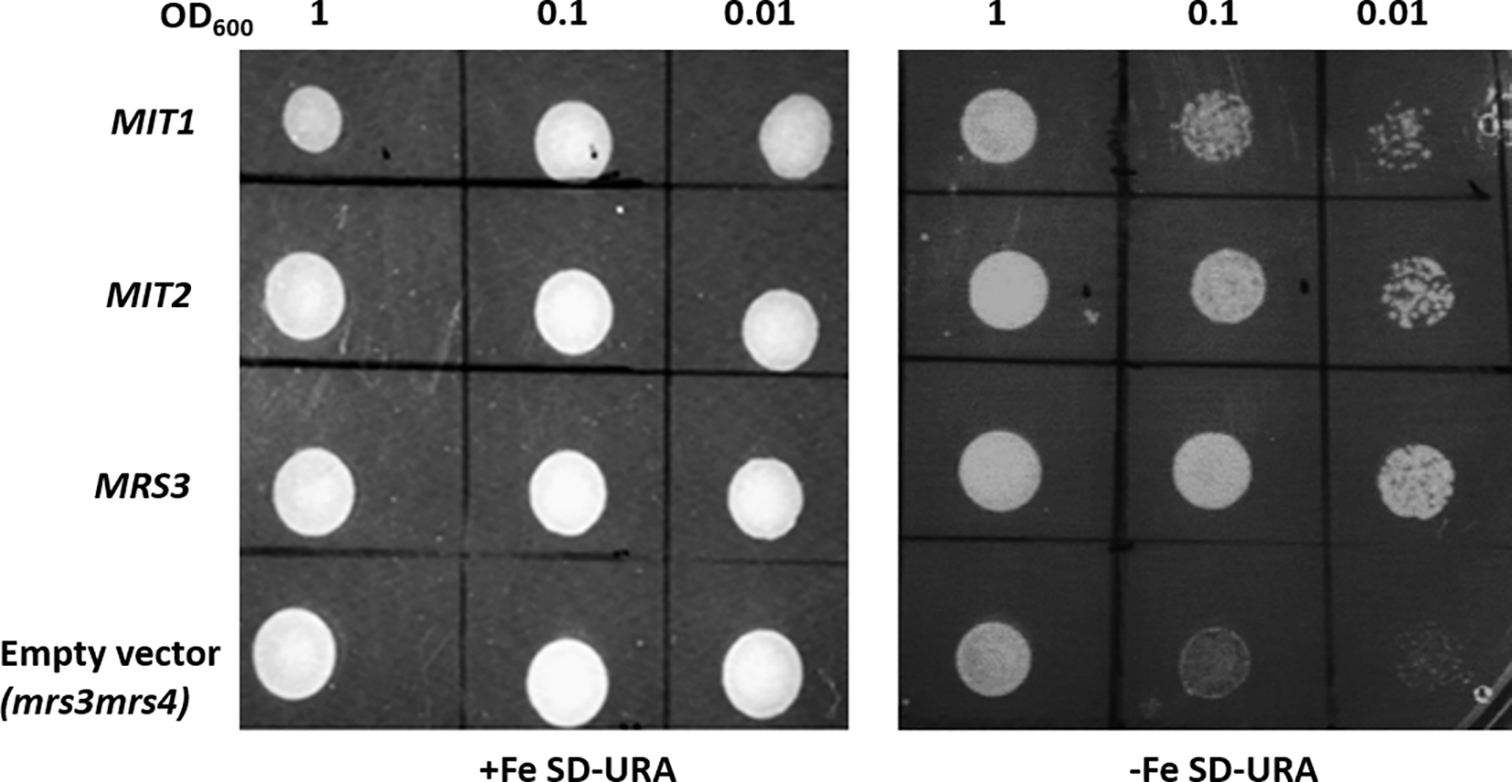
Arabidopsis MIT1 and MIT2 complement the defective growth phenotype of *mrs3mrs4* on Fe-deficient media. *mrs3mrs4* was transformed with *MIT1, MIT2, MRS3* (positive control) and the empty vector (pRS426-ADH; negative control). Three serial dilutions corresponding to OD_600_ of 1, 0.1 and 0.01 of the transformed yeast were assayed for growth complementation on SD-uracil plates under Fe-sufficient (0.1mM FeSO_4_) or Fe-deficient (0.1mM BPS) conditions.

The yeast genes, *MRS3* and *MRS4* encode functional mitochondrial iron transporters that mediate the uptake of Fe^2+^ to the mitochondria. The double mutant, *mrs3mrs4* exhibits a growth sensitive phenotype under low Fe conditions (Muhlenhoff et al., 2003). To test if Arabidopsis MIT1 and MIT2 are functional orthologs of MRS3 and MRS4, we cloned *MIT1* and *MIT2* separately into a yeast expression vector (pRS426-ADH) and transformed them into the *mrs3mrs4* yeast background to assess their ability to complement the poor growth phenotype of the mutant strain. To ensure proper targeting of the Arabidopsis proteins to the yeast mitochondria, the endogenous targeting sequences (1st-22 amino acids) of MIT1 and MIT2 were substituted with the mitochondrial leader sequence (1st-22 amino acids) of MRS3 (as employed in Shaw et al., 2006). The yeast *MRS3* clone and the empty vector were used as positive and negative controls, respectively. The *mrs3mrs4* strain transformed with *MIT1*, *MIT2*, *MRS3*, or the empty vector. The resulting strains were spotted side-by-side on Fe-sufficient and Fe-deficient media. No phenotypic differences were observed between the different strains under Fe sufficiency. Interestingly, similar to yeast MRS3, both MIT1 and MIT2 were able to partially rescue the slow growth phenotype of *mrs3mrs4* under Fe deficiency (**Figure 2**). In contrast, the empty vector strain was unable to grow well on Fe-deficient media. These results show that Arabidopsis MIT1 and MIT2 can rescue the loss of function phenotype of mitochondrial iron transporters in yeast. Differences in the efficiency of complementation by MIT1 and MIT2 could be due to expression and/or targeting of the Arabidopsis proteins in yeast.

### MIT1 and MIT2 Localize to Mitochondria in Plants

**Figure 3:**
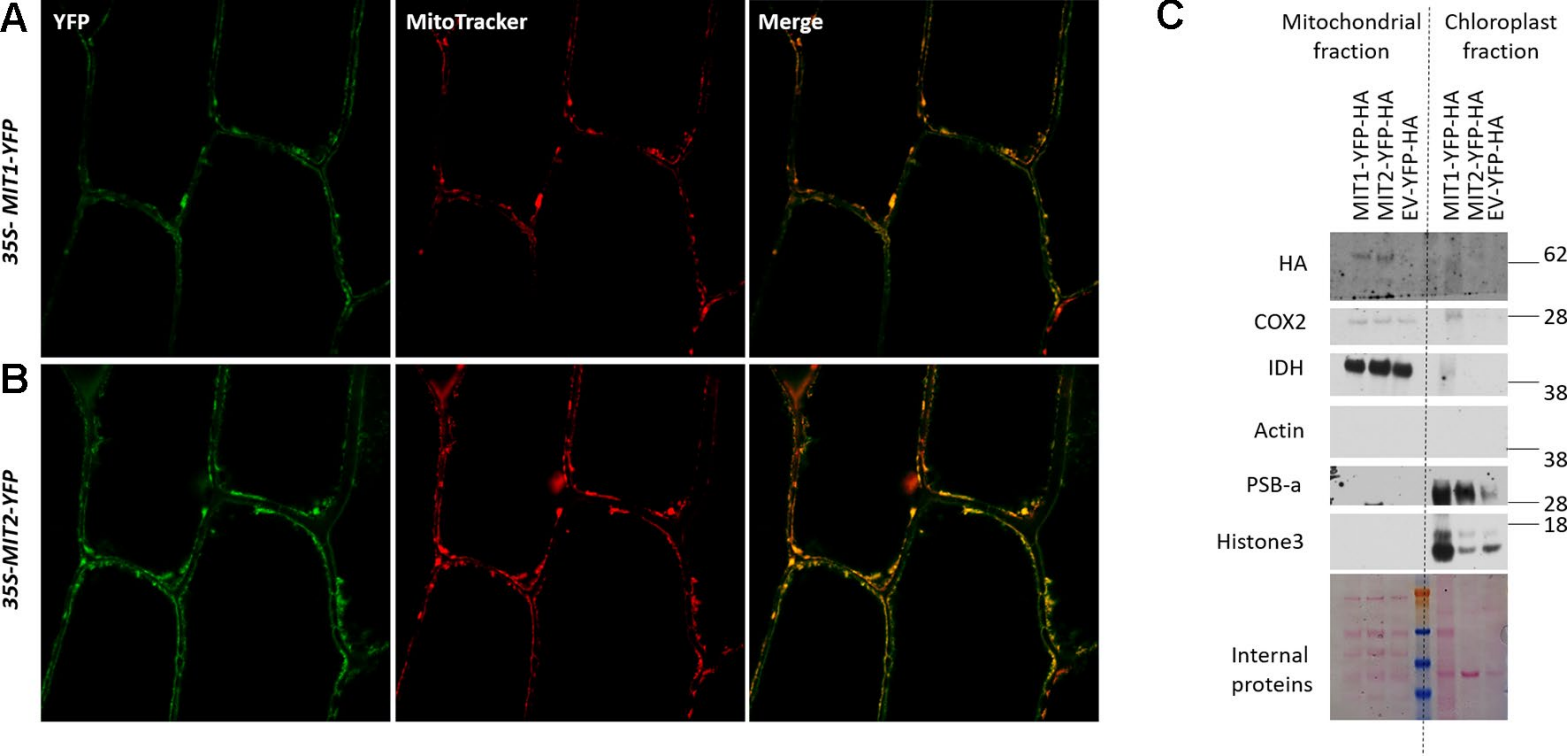
Mitochondrial localization of MIT proteins. Confocal images of onion peel epidermis transiently transformed with **(A)** *35S-MIT1-YFP* and **(B)** *35S-MIT2-YFP* constructs. The cells were co-stained with the mitochondria specific marker, MitoTracker Orange. **(C)** Mitochondria were purified from 2-week-old Arabidopsis*MIT1-YFP-HA*, *MIT2-YFP-HA* and the empty vector (*EV-YFP-HA*) transgenic lines grown on B5 media. 25 µg of the purified protein was separated by the SDS-PAGE. The localization of MIT1 and MIT2 was tested by immunoblotting with an anti-HA antibody. The chloroplast fraction (comprised of broken thylakoids/chloroplasts) separated from the mitochondrial fraction during ultracentrifugation were used as a control. The purity of the mitochondria was tested by immunoblotting the purified mitochondria against different cellular markers including COX2 and IDH (mitochondrial proteins), Actin (cytosolic protein), PSB-a (chloroplast protein) and Histone3 (nuclear protein). Ponceau S-stained proteins were used as a loading control. Note: the chloroplast MIT1-YFP-HA lane is overloaded relative to the MIT2-YFP-HA and EV-YFP-HA lanes.

MIT1 and MIT2 have been previously predicted to localize to the mitochondria due to their possession of the METS (Millar and Heazlewood, 2003). To investigate their subcellular localization *in planta,* the *MIT1* and *MIT2* clones, driven by the *35S* promoter were fused in frame with a YFP tag (*35S-MIT1-YFP* and *35S-MIT2-YFP*) and these constructs were transiently transformed into onion peel epidermis *via* particle bombardment. The transformed epidermis peels were co-stained with a mitochondrial marker (MitoTracker Orange) and were further analyzed for fluorescence using confocal microscopy. Both MIT1 and MIT2 colocalized with the mitochondrial marker thus confirming their localization to mitochondria (**Figures 3A, B**). To further confirm the localization, *35S-MIT1-YFP* and *35S-MIT2-YFP* stable transgenic Arabidopsis lines along with the empty vector line were also generated. We purified the mitochondria from these lines and assessed the localization of MIT1 and MIT2 *via* western blot. The purity of the mitochondria was analyzed by probing against various known markers of other subcellular compartments. These studies confirmed the localization of both proteins to the mitochondria (**Figure 3C**). In addition, it is important to note that C-terminally tagged MIT1 and MIT2 were able to rescue the yeast *mrs3mrs4* mutant, so presumably, these tags do not disrupt transporter function.

### Expression Pattern Analysis of *MIT1* and *MIT2*

**Figure 4:**
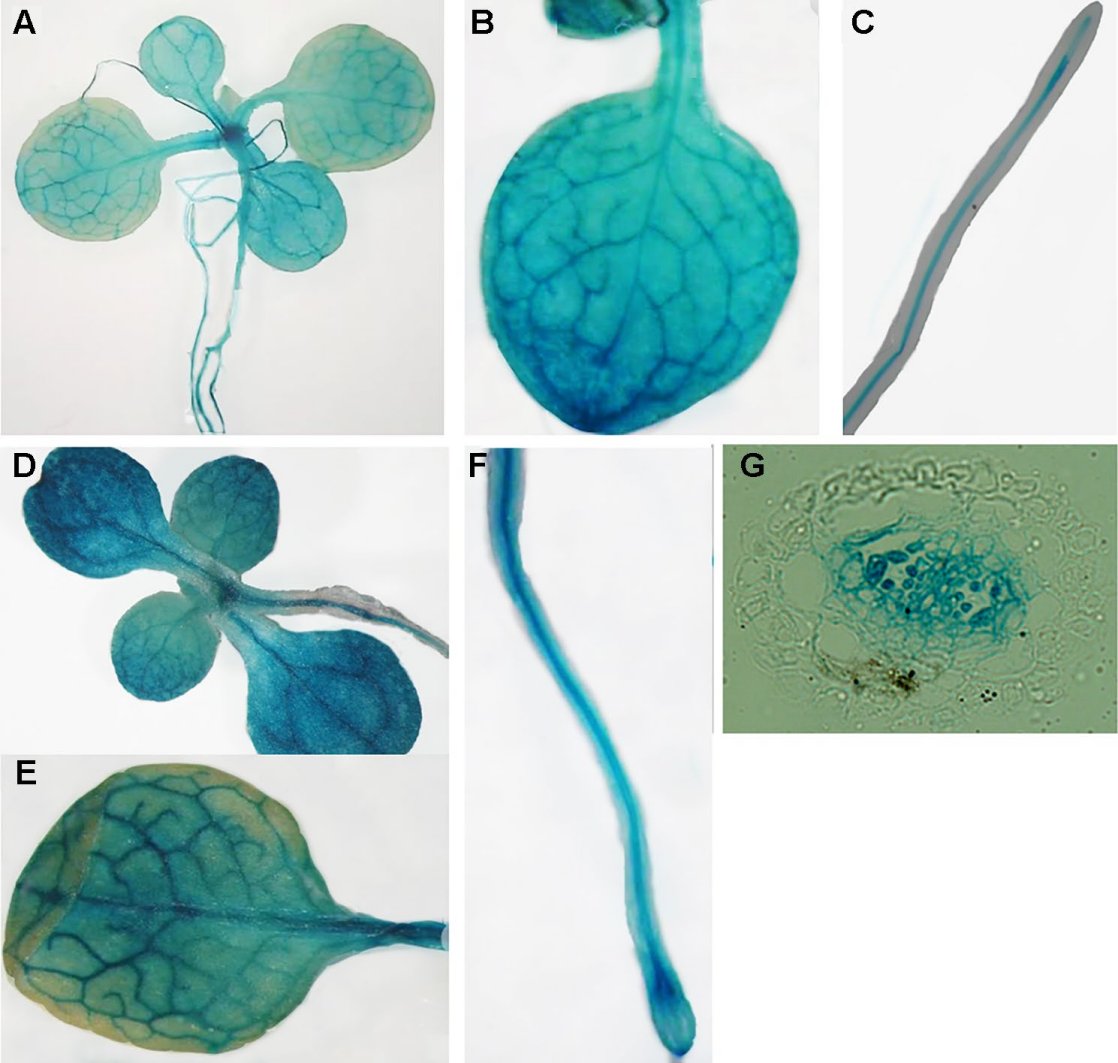
Expression analysis of *MIT1* and *MIT2*. GUS histochemical staining of 2-week-old seedlings grown in B5 media. **(A**-**C)** *pMIT1-GUS* and **(D**-**F)** *pMIT2-GUS*. **(G)** Root cross-section of *pMIT2-GUS*.

Publicly-available gene expression data shows that *MIT1* and *MIT2* are expressed throughout development. However, the expression of both genes is the highest in the seed and young developing seedlings (http://bar.utoronto.ca/efp/cgi-bin/efpWeb.cgi). To study the spatial expression patterns of *MIT1* and *MIT2,* we generated stable transgenic lines transformed with β-glucuronidase (GUS) reporter constructs driven by either the *MIT1* or *MIT2* endogenous promoters. GUS histochemical staining was performed on 2-week-old seedlings, and the staining was observed in both shoots and roots of the young seedlings of *pMIT1-GUS*, as well as *pMIT2-GUS* (**Figures 4A–G**). These results confirm that *MIT1* and *MIT2* are ubiquitously expressed. In addition, the promoter activity for both genes is notably pronounced in the vascular cylinder of the plant as previously observed (Dinneny et al., 2008). In fact, *MIT1* and *MIT2* were identified as genes whose expression is particularly high in the pericycle (Long et al., 2010).

**Figure 5:**
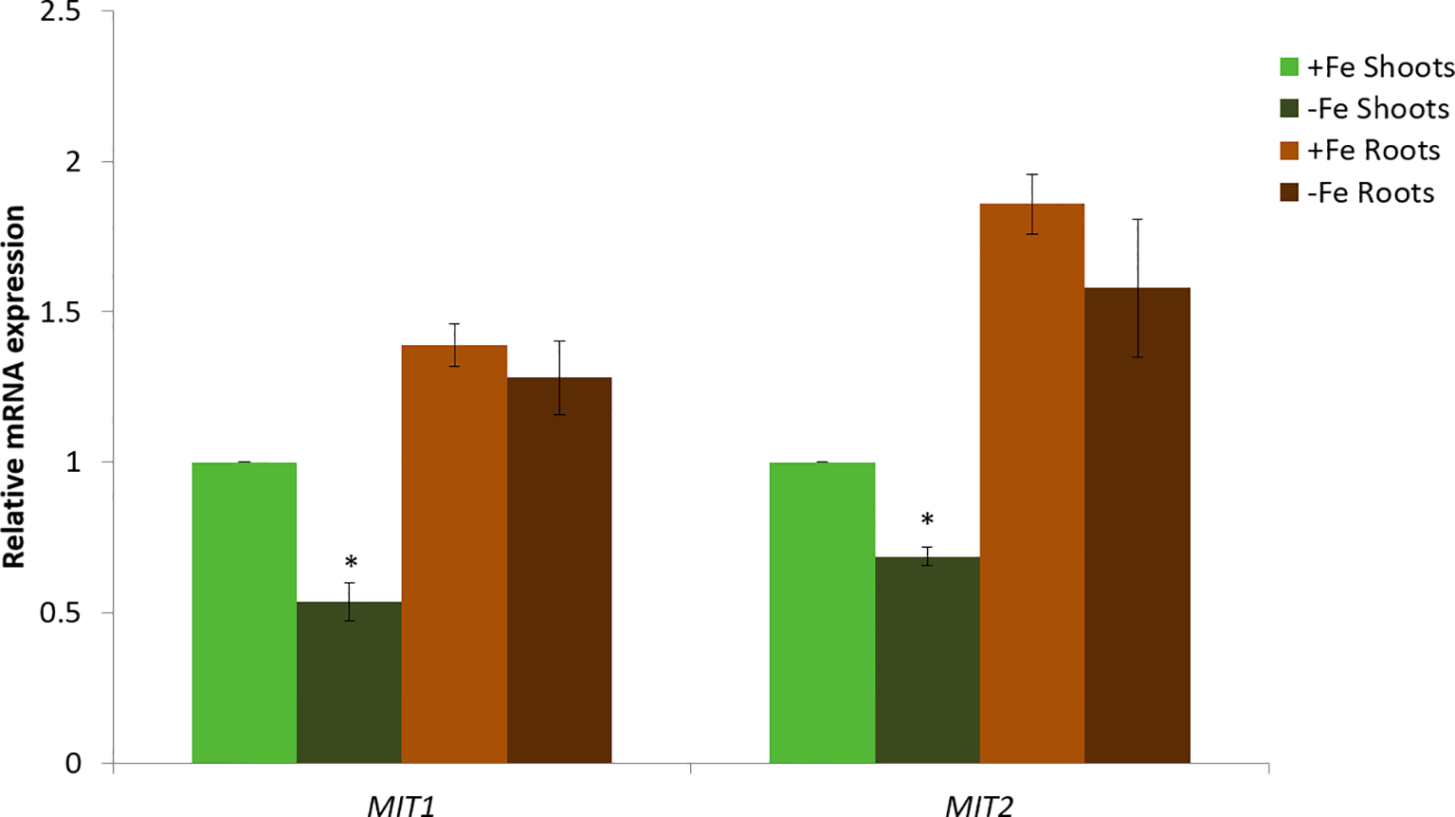
Quantitative RT-PCR expression analysis of *MIT1* and *MIT2* in shoots and roots of WT seedlings grown in standard B5 media for 2 weeks and then transferred to Fe-sufficient or Fe-deficient media for 3 days. The mRNA levels for both *MIT1* and *MIT2* from +Fe shoot was set at 1 and the relative abundance of the two transcripts were determined in the other tissues. Actin was used as a reference gene to normalize mRNA values. Values represent the mean of 3 biological replicates, and error bars indicate standard deviation. Significance (p < 0.05) was assessed using the Student’s *t*-test; significant differences in the transcript abundance between +Fe and −Fe shoots is represented by an asterisk.

Next, we looked at the expression levels of *MIT1* and *MIT2* in the roots and shoots of Col-*0* (WT) seedlings under Fe-deficient and Fe-sufficient conditions by quantitative RT-PCR (**Figure 5**). Both the genes are expressed in shoots as well as roots. Both *MIT1* and *MIT2* show a modest decrease in mRNA levels upon Fe limitation in shoots but no response to Fe limitation in the roots of 2-week-old seedlings. Thus, our data indicate that unlike rice *MIT*, the expression of *MIT1* and *MIT2* in Arabidopsis is not strongly regulated by Fe availability. Similar results were observed previously in microarray analysis performed on Arabidopsis seedlings grown under +/-Fe conditions (Long et al., 2010). This data is supported by RNA sequencing of the *nramp3nramp4* line (which is defective in Fe remobilization from vacuoles) (Bastow et al., 2018). While the other markers of Fe-deficiency such as putative mitochondrial Fe reductase *FRO3*, the root epidermis Fe reductase *FRO2* and the root epidermis Fe transporter *IRT1* were upregulated in *nramp3nramp4,* expression of *MIT1* and *MIT2* is unaffected in the same background (Bastow et al., 2018). Thus, it appears that MIT expression is not strongly regulated by Fe status in Arabidopsis.

### MIT1 and MIT2 Are Essential for Embryogenesis

**Figure 6:**
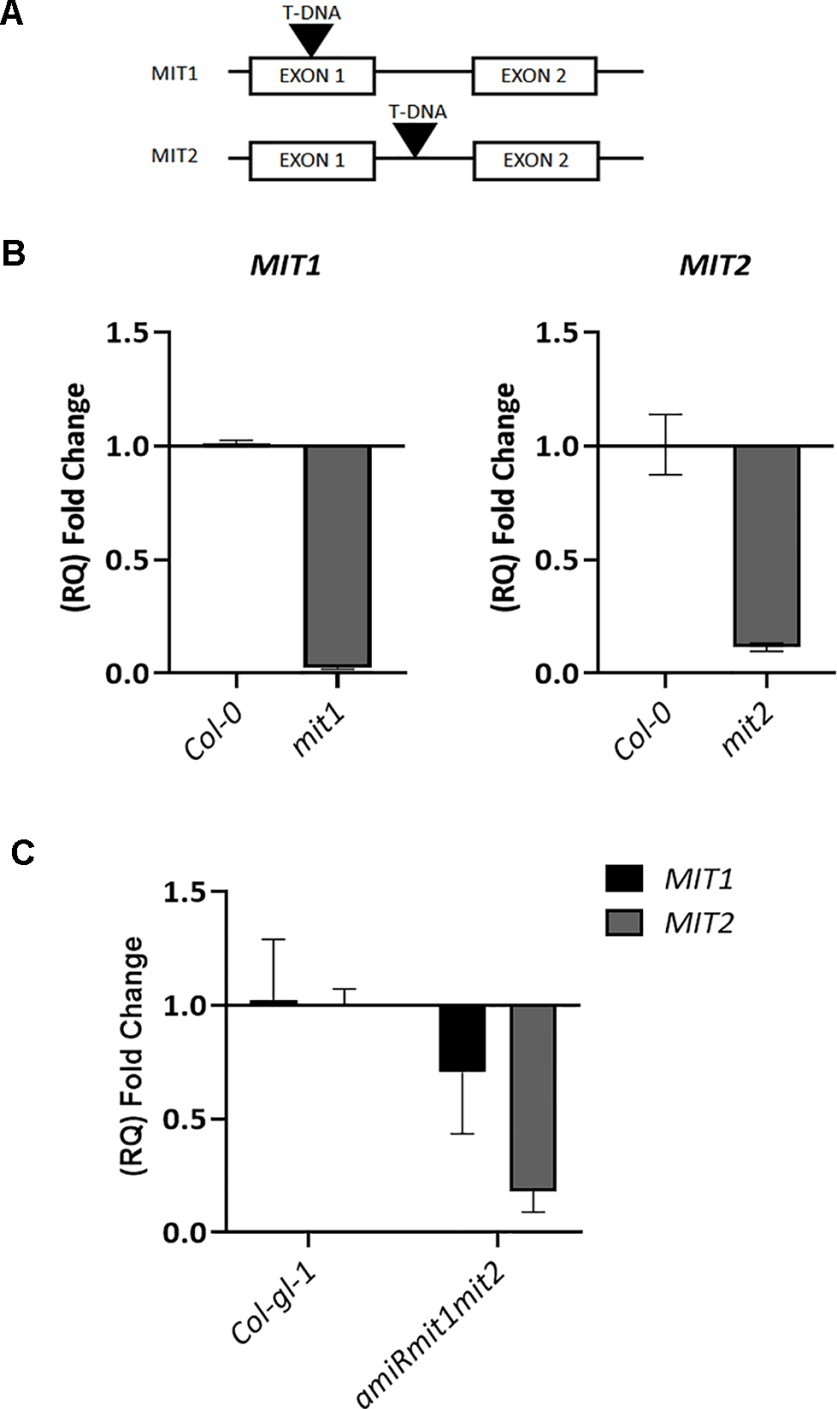
Genetic analysis of *mit1* and *mit2* mutants. **(A)** Gene structure of the *mit1* and *mit2* T-DNA insertion mutants. The insertion in *mit1* was identified in its first exon, 246 bases after the translation start site, the insertion in *mit2* is located in the intron, 1592 bases after the translation start site. **(B)** Relative quantification (RQ) of *MIT1 and MIT2* transcript levels by quantitative RT-PCR in *mit1* and *mit2*, respectively. **(C)** Relative quantification (RQ) of *MIT1* and *MIT2* in Col-*gl-1* and *amiRmit1mit2* lines. Actin was used a control. The values represent the mean of two to three biological replicates, and error bars indicate standard deviation. The RNA was harvested from 2.5-week-old seedlings grown in Fe-sufficient media.

To investigate the role of *MIT1* and *MIT2 in planta*, T-DNA insertion mutants (SALK_013388 for *MIT1* and SALK_096697 for *MIT2*) were obtained from the ABRC. *MIT1* (At2g30160) is 1862 bp long with two exons. *MIT2* (At1g07030) is a 2405 bp long gene with two exons and a single intron each. While the *mit1* mutant (SALK_013388) has an insertion in its first exon, 246 bases after the translation start site, the *mit2* (SALK_096697) mutant has a single T-DNA insertion in the intron, 1592 bases after the translation start site (**Figure 6A**). Single-insertion homozygous mutants, (*mit1* and *mit2*) were confirmed by back-crossing the mutants with the WT and PCR genotyping. The heterozygous F1 generation obtained after backcrossing was allowed to self-cross which resulted in a progeny population of 1:2:1 (WT: heterozygous: homozygous) in the following generation (F2). These lines were grown and genotyped for three more generations to ensure a single insertion. The true breeding lines thus obtained were propagated as homozygous single insertion. Transcript levels in the mutants were measured by quantitative and semi-quantitative RT-PCR (**Figures 6B** and **S1A**). The *mit1* insertion resulted in an almost complete knockout with 98% reduction in the *MIT1* transcript abundance while the insertion in the *mit2* line resulted in 85% knockdown of *MIT2* transcript abundance (**Figure 6B**).

**Figure 7:**
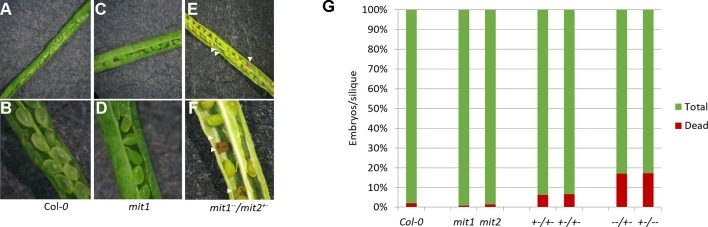
*mit1mit2* mutation is embryonically lethal. Dissected siliques of **(A**, **B)** WT, **(C**, **D)** *mit1*, **(E**, **F)** *mit1* ^−−^ */mit2* *^+–^*. The aborted embryos (red embryos and empty spaces in the silique; indicated by white arrows) show the embryonic lethality of *mit1mit2* homozygous mutants. **(G)** Quantification of aborted embryos in different *mit* mutant backgrounds. *mit1* ^−−^ */mit2* *^+–^* is represented as ^−−^/*^+^* ^−^ and *mit1* *^+^* ^−^ */mit2* ^−−^ is represented as +−/−−. Approximately 500 seeds were counted from 10 to 12 individual siliques per genotype.

The visible phenotypes of the single mutants were indistinguishable from the wild type (data not shown). We therefore hypothesized that MIT1 and MIT2 function redundantly, and so the individual *mit1* and *mit2* T-DNA insertion lines were crossed together to obtain a double knockout line *mit1mit2*. However, we failed to obtain a line homozygous for both *mit1* and *mit2.* Dissection of the siliques of self-crossed *mit1* ^−−^ */mit2* *^+^* ^−^ revealed an embryo lethal phenotype presumably due to the loss of both *MIT1* and *MIT2* (**Figures 7A–F**). Similar results were observed by self-crossing the *mit1* *^+^* ^−^ */mit* ^−−^ line (image not shown). 6.6% of embryos were aborted in the heterozygous *mit1* ^−−^ */mit2* *^+^* ^−^, while 20.4% and 20.8% of embryos were aborted in the self-crossed lines *mit1* ^−−^ */mit2* *^+^* ^−^ and *mit1* *^+^* ^−^ */mit2* ^−−^ respectively (**Figure 7G**). The statistical significance of these ratios was confirmed by chi-square test (chi-square value <3.84). The fact that the *mit2* mutation does not result in a full knockout may account for the fact that we observed less than the predicted 25% embryo lethality. These results demonstrate that the two genes are functionally redundant and MIT function is essential for embryogenesis. All further experiments for the functional analysis of MIT function were performed using the *mit1* ^−−^ */mit2* *^+^* ^−^ line due to a stronger transcript suppression in this line as compared to *mit1* *^+^* ^−^ */mit2* ^−−^ (**Figure S1B**).

Artificial microRNA lines targeting both *MIT1* and *MIT2* (*amiRmit1mit2)* were constructed to confirm the double mutant phenotypes. Five independent lines were tested, and the one (A17) with the lowest transcript abundance (with a 30% reduction in *MIT1* and 88% reduction in *MIT2* levels) of *MIT1* and *MIT2* was chosen for all further experiments (**Figure 6C**). Due to only a partial reduction of transcript abundance, the *amiRmit1mit2* mutant did not exhibit an embryo lethal phenotype but it was subject to further phenotypic profiling (along with *mit1* ^−−^ */mit2* *^+^* ^−^ *)* as described below.

### MIT1 and MIT2 Mediate Mitochondrial Iron Uptake in Arabidopsis

**Figure 8:**
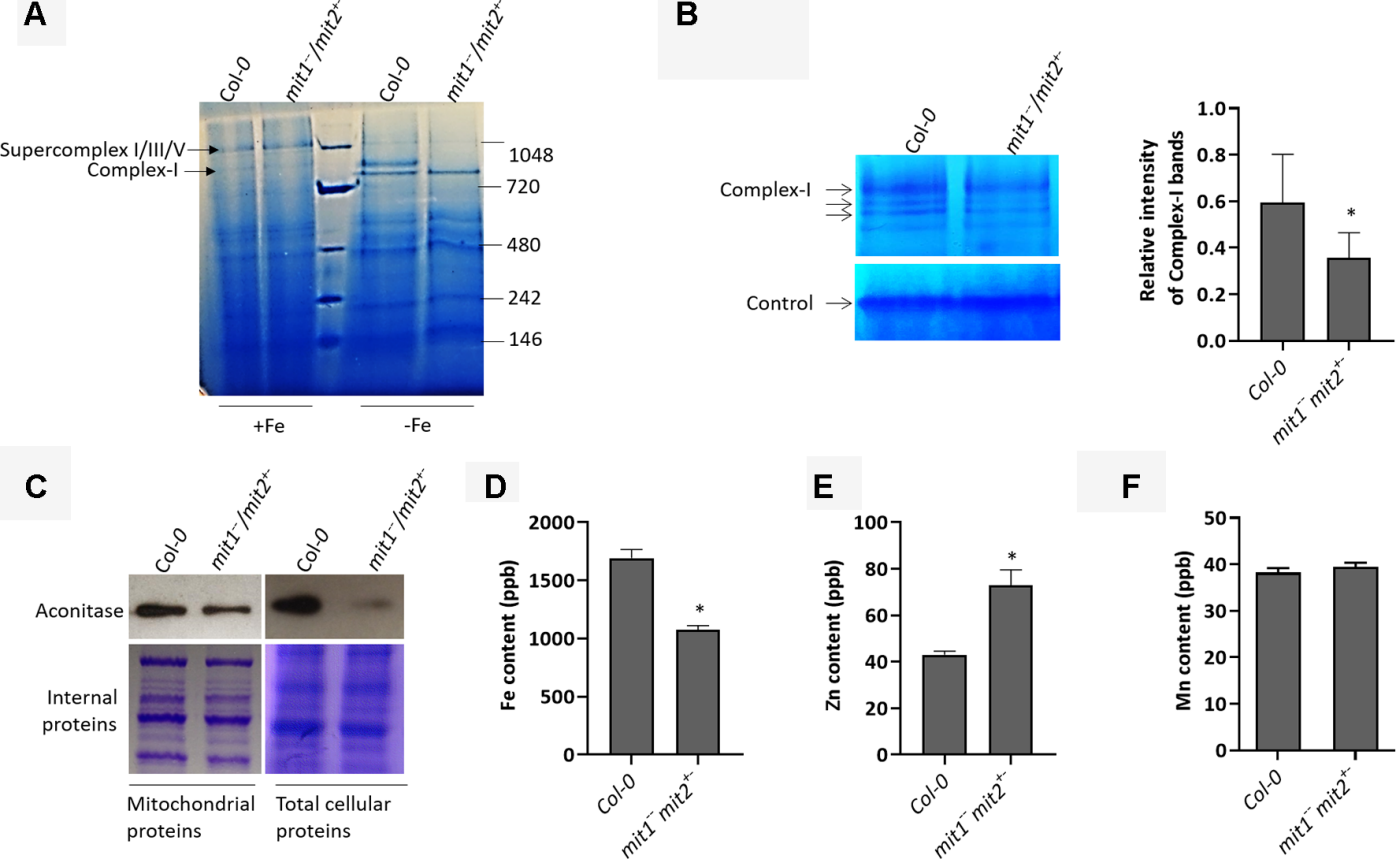
MITs are important for mitochondrial iron acquisition/import. **(A)** Separation of 20 µg solubilized mitochondrial fraction of Col-*0* and the *mit1* ^−−^ */mit2* *^+^* ^−^ mutant grown in Fe-sufficient (+Fe) or Fe drop-out media (-Fe) by blue-native gel electrophoresis (BN-PAGE). **(B)** In-gel enzyme activity assay for complex I on mitochondrial extract separated by BN-PAGE. The arrows indicate complex I and its supercomplexes. Non-specific staining at the bottom of the gel was used as the loading control. The staining in the WT and the mutant was quantified using ImageJ software. **(C)** Immunoblots showing aconitase protein levels in mitochondrial and total protein extracts. Coomassie stained internal proteins were used as a loading control. Total mitochondrial **(D)** Fe content, **(E)** Zn content, and **(F)** Mn content as measured by ICP-MS (shown is the mean of three technical replicates; error bars represent standard deviation). Significance was assessed using the student’s *t*-test. An asterisk represents a p value < 0.05. The mitochondria for all the experiments **(B**-**F)** were isolated from 2.5-week-old seedlings grown on Fe drop-out media.

Yeast mitoferrins are believed to function predominantly under low Fe conditions (Froschauer et al., 2009). To study the role of MIT1 and MIT2 in mitochondrial Fe homeostasis, we purified mitochondria from the WT and the *mit1* ^−−^ */mit2* *^+^* ^−^ mutant grown in Fe-sufficient and Fe drop-out media. It is important to note that the mitochondria prepared from *mit1* ^−−^ */mit2* *^+^* ^−^ were obtained from a population that was homozygous for the *mit1* mutation but was segregating for *mit2*. Since several respiratory subunits require Fe-S clusters and/or heme as their cofactor, Fe-limitation is consequently expected to affect the integrity and the function of the electron transport chain in mitochondria. Therefore, we first investigated the effect of loss of MIT1 and MIT2 on the respiratory complexes by separating solubilized mitochondrial proteins on a blue native gel. While no significant difference was observed between the WT and *mit1* ^−−^ */mit2* *^+^* ^−^ mutant mitochondria obtained from Fe-sufficient conditions, significant changes in the relative abundance of the complexes and supercomplexes close to 1 megadalton (typically complex I and its supercomplexes) were observed in the *mit1* ^−−^ */mit2* *^+^* ^−^ mutant mitochondria isolated from plants grown in Fe drop-out media (**Figure 8A**). To confirm, we performed in-gel staining for complex I and found a 40% reduction in complex I levels in the mutant mitochondria (**Figure 8B**). A 30.2% reduction in complex I was also observed in the *amiRmit1mit2* mutant mitochondria isolated from seedlings grown in Fe drop-out conditions (**Figure S2A**). We also examined the protein levels of aconitase, a [4Fe-4S] cluster protein, involved in the TCA cycle in mitochondria. Previous studies have shown that Fe deficiency results in reduced aconitase abundance (Ross and Eisenstein, 2002). As expected, reduced aconitase protein levels were observed in the mitochondrial as well as total cellular extracts of *mit* mutants grown in the Fe drop-out conditions (**Figure 8C**). Since the loss of MIT1 and MIT2 had a more pronounced effect in Fe-limited conditions, we measured the elemental profile of mitochondria prepared from 2.5-week-old WT and *mit1* ^−−^ */mit2* *^+^* ^−^ seedlings grown on Fe drop-out media. The Fe levels in mitochondrial preparations of the *mit1* ^−−^ */mit2* *^+^* ^−^ mutant were significantly reduced (by 43%) as compared with WT (**Figure 8D**). The *amiRmit1mit2* lines also show a reduction in mitochondrial Fe content (**Figure S2B**). Interestingly, these mutants also show an increased accumulation of Zn in their mitochondria (**Figures 8E** and **S2B**), suggesting cross-talk between Fe and Zn homeostasis. The Mn content of the mitochondria was unchanged (**Figures 8F** and **S2C**), and the levels of Cu and Co were below the detection limit. These results indicate that in the absence of MIT1 and MIT2, mitochondrial iron import is severely compromised resulting in significant loss of Fe-requiring proteins and their biochemical activities. Moreover, the loss of MIT1 and MIT2 appears to be significant for mitochondrial function primarily under Fe drop-out conditions, suggesting the possible existence of an alternate Fe uptake pathway in mitochondria.

### *mit* Mutants Display Altered Whole-Plant Iron Homeostasis

**Figure 9:**
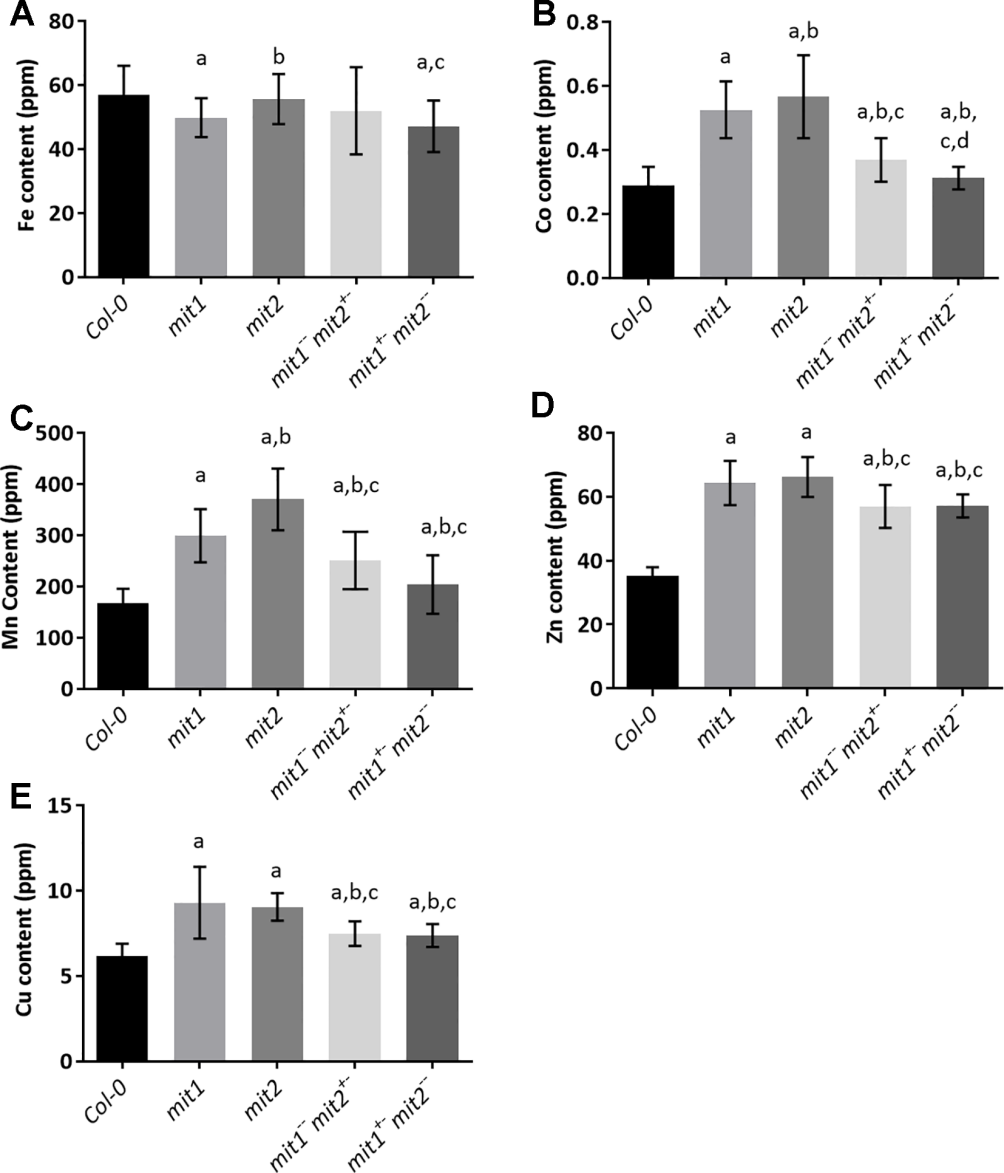
*mit* mutants exhibit iron deficiency. Levels of **(A)** Fe, **(B)** Co, **(C)** Mn, **(D)** Zn, **(E)** Cu in the shoots of plants grown in soil in short day conditions for 44 days. Values shown are an average of 13 biological replicates and error bars represent standard deviation. Significance was assessed using the Student’s *t*-test. A p value < 0.05 compared to Col*-0*, *mit1*, *mit2*, *mit1* *^--^* */mit2* *^+-^* is represented by a, b, c, d respectively.

To gain insight into the effect of loss of MIT function on iron homeostasis at the whole plant level, we measured the elemental profile of the shoots of 44-day-old mature, soil grown plants (WT, *mit1, mit2,* *mit1* ^−−^ */mit2* *^+^* ^−^ *, mit1* *^+^* ^−^ */mit2* ^−−^, and *amiRmit1mit2*) by ICP-MS. Shoot Fe levels showed slight to no change in the mutant lines as compared to the WT (**Figures 9A** and **S2D**). However, all the mutants exhibited the well-described Fe deficiency signature phenotype (Baxter et al., 2008) as shown by elevated levels of Mn, Zn, and Co observed in the shoots of the *mit1, mit2* and the double mutants (**Figures 9B–D**). Additionally, these mutants also accumulated significant amounts of Cu (**Figure 9E**). The elemental profile observed in *mit* lines suggests that these lines perceive Fe deficiency, which in turn results in increased uptake of Zn and other divalent metals potentially *via* IRT1, although Fe levels remain relatively stable, likely due to tight control on Fe homeostasis to prevent its overaccumulation.

**Figure 10:**
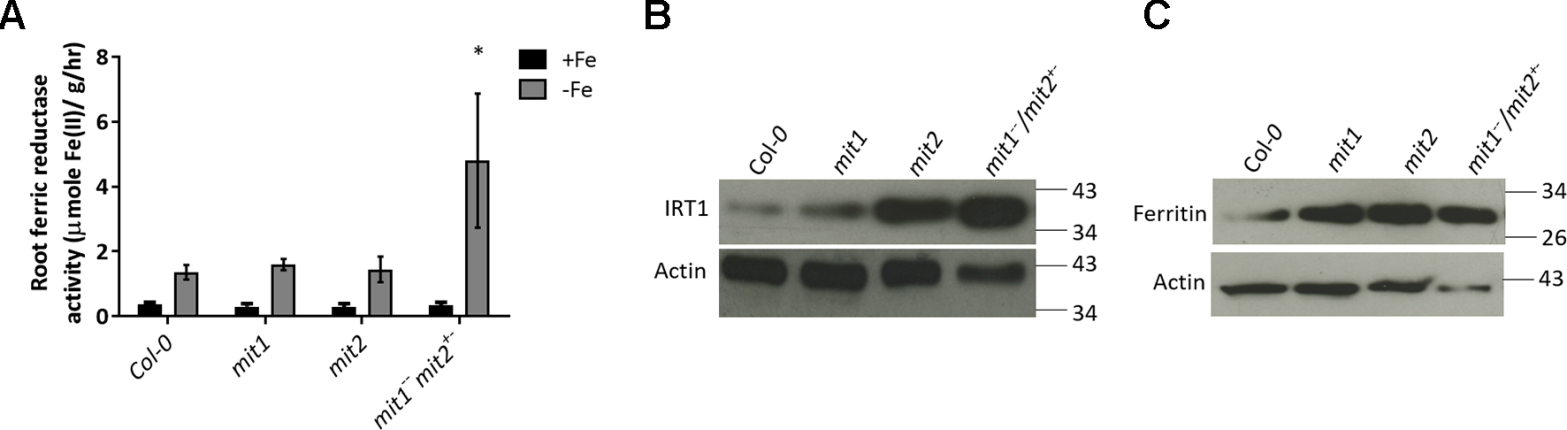
*mit* mutants exhibit altered Fe homeostasis under Fe deficiency. **(A)** Root ferric reductase activity assay on seedlings grown in Fe-sufficient or Fe-deficient media. The activity was normalized to root fresh weight. Values shown are an average of 10 biological replicates. Error bars in all the graphs indicate standard deviation. Significance was assessed using the student’s *t*-test. The asterisk represents a p < value 0.05 as compared to Col-*0.* Seedlings were grown on standard B5 media for 2 weeks and then transferred to Fe-sufficient (50 µM Fe(III)-EDTA) or Fe-deficient (300 µM ferrozine media) for 3 days. **(B)** Immunoblot of IRT1 protein levels in the roots of *mit* mutants grown on standard B5 media for 2 weeks and then transferred to Fe-deficient conditions for 3 days. **(C)** Immunoblot of ferritin levels in shoots of 2.5-week-old *mit* seedlings grown in Fe-sufficient media. 25 µg of protein lysate was loaded per lane for the immunoblots.

To test the hypothesis that loss of MIT function results in upregulation of Fe deficiency responses, we measured the levels of two root Fe deficiency markers. First, we measured root surface ferric reductase activity and showed that while ferric reductase activity is not altered in the single *mit1* and *mit2* mutants, the *mit1* ^−−^ */mit2* *^+^* ^−^ and *amiRmit1mit2* lines display significant increases in induction of ferric reductase activity under Fe deficiency as compared to WT (**Figures 10A** and **S2E**). Similarly, IRT1 protein levels are also significantly higher in the mutant lines (**Figures 10B** and **S2F**). Furthermore, we also observed a significant elevation in ferritin levels in *mit* mutant shoots (**Figure 10C**) in Arabidopsis supporting the hypothesis that Fe homeostasis is disrupted in *mit* plants.

**Figure 11:**
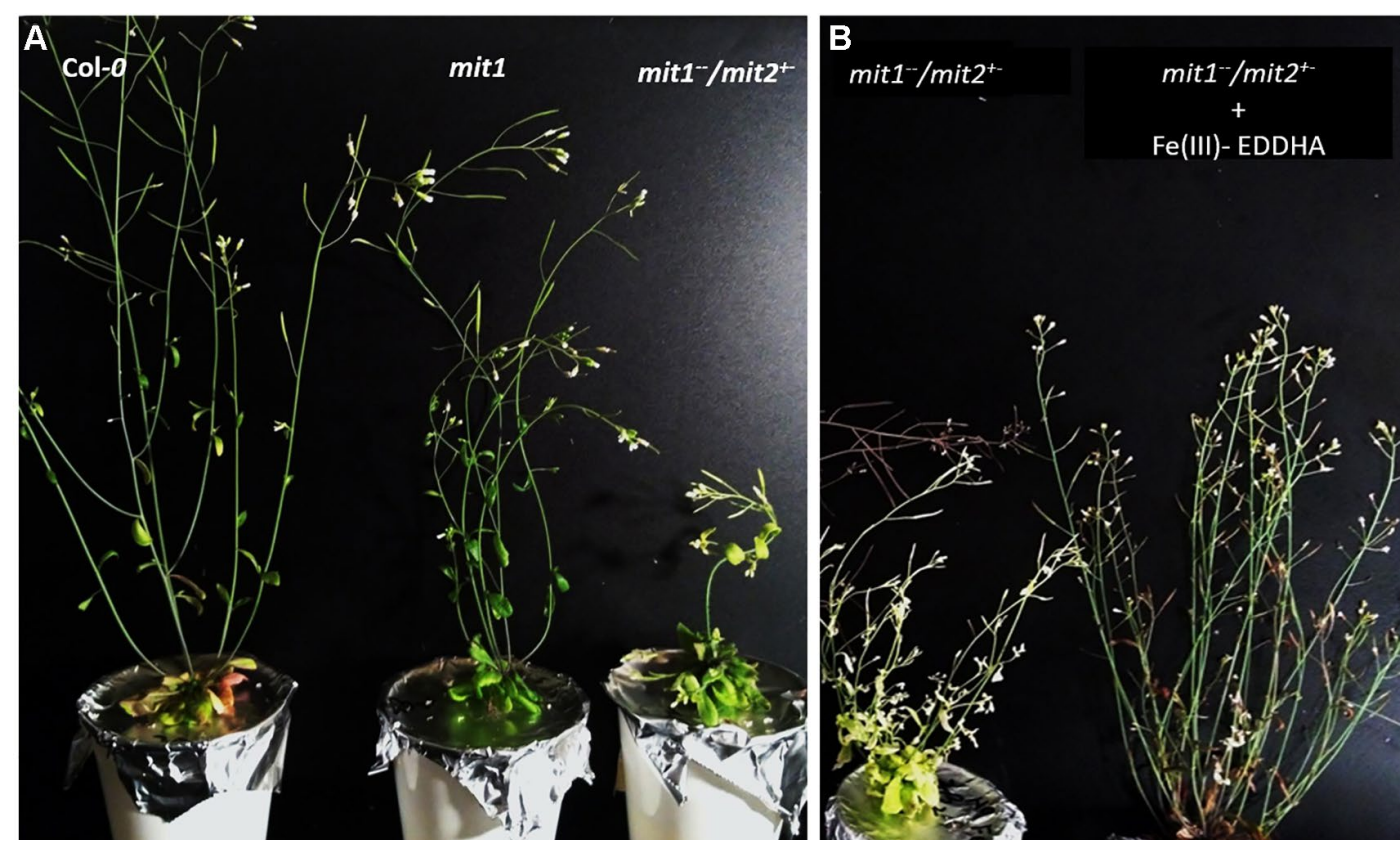
Growth phenotype of mit loss-of-function lines in Fe-deficient conditions. **(A)** Compromised growth phenotype of five-week old *mit1* ^−−^ */mit2* *^+^* ^−^ grown hydroponically in Fe drop-out media. **(B)** Six-week old plants grown hydroponically in Fe drop-out media supplemented with 5 µM Fe (III)-EDDHA after 3 weeks.

In addition to these molecular phenotypes, we also observed the general growth and development of *mit* loss of function lines. While the soil grown mutants did not show any visible phenotypic differences from the WT, *mit* mutants lines appeared to be chlorotic and displayed a compromised growth phenotype when grown hydroponically in Fe drop-out media (**Figures 11A** and **S3**). This phenotype could be rescued *via* exogenous supply of 5 µM Fe(III)-EDDHA, suggesting that MIT loss-of-function phenotypes are due to altered Fe metabolism (**Figure 11B**).

## Discussion

Mitochondrial Fe is generally known to be required for two major biochemical pathways: Fe-S cluster biogenesis and heme synthesis. Despite its importance, Fe transport across the mitochondrial membrane is still not fully understood. Our studies in Arabidopsis have identified two putative mitochondrial metalloreductases (FRO3 and FRO8), of which FRO3 is hypothesized to play a role in mitochondrial iron transport based on the fact that it can reduce Fe in a heterologous system (Wu et al., 2005), is broadly expressed and is upregulated by Fe limitation (Mukherjee et al., 2006;Jain et al., 2014). Although proteins involved in Fe export from plant mitochondria remain unidentified, a recent study has shown that ATM3 (a member of the ATP Binding Cassette (ABC) family) exports glutathione polysulphide for the assembly of cytosolic Fe-S clusters in Arabidopsis (Schaedler et al., 2014). In this paper, we provide evidence that yeast MRS3 and MRS4 orthologs, MIT1 and MIT2 function redundantly as mitochondrial iron transporters in Arabidopsis.

Mitochondrial carrier family (MCF) proteins are small 30kDa proteins that localize to the inner mitochondrial membrane (IMM) and are involved in the transport of a wide variety of solutes from the inner membrane space (IMS) into the mitochondrial matrix (Walker, 1992; Palmieri, 2013). The outer mitochondrial membrane (OMM) possesses porin proteins which allow the free movement of various small molecules across the membrane. The IMM, on the other hand, is selectively permeable, and therefore requires transporters to shuttle polar solutes across the membrane. MCF proteins have a tripartite structure, with six transmembrane domains forming a barrel for substrate transport, which is closed at the matrix side by a salt-bridge network that is located at the bottom of the cavity (Pebay-Peyroula et al., 2003; Kunji and Robinson, 2006). Arabidopsis MIT1 and MIT2 belong to the MCF protein family and therefore likely reside at the IMM of mitochondria (Picault et al., 2004). A recent report described amino acid residues of Mfrn1 and MRS3 that are important for Fe transport across the membrane (Brazzolotto et al., 2014; Christenson et al., 2018). The presence of these residues in the Arabidopsis MIT1 and MIT2 amino acid sequences (Figure 1) support their roles as mitochondrial Fe transporters (Kunji and Robinson, 2006; Brazzolotto et al., 2014; Connorton et al., 2017; Christenson et al., 2018). Expression of *35S-MIT-YFP* constructs in onion peel epidermal cells allowed us to show that MIT1 and MIT2 are indeed localized to mitochondria (**Figure 3**). The functional role of MIT1 and MIT2 as mitochondrial Fe transporters was confirmed by complementation of the yeast mitoferrin mutant (*mrs3mrs4*) (**Figure 2**).

To better understand the molecular function of *MIT1* and *MIT2 in planta*, we identified T-DNA insertion mutants of *MIT1* and *MIT2* (**Figure 6**). The redundancy in the roles of mitoferrins has been previously observed in mammalian non-erythroid cells, as well as yeast in terms of biochemical properties and kinetic profiles for Fe^2+^ uptake (Paradkar et al., 2009; Brazzolotto et al., 2014). While the double mutant was embryo lethal, no obvious growth defects were noted when the single mutants were grown in soil or on MS agar plates (data not shown). Similar to the *mit1mit2* double mutant, the frataxin mutant (*atfh*) also exhibits an embryo lethal phenotype (Vazzola et al., 2007). Despite the accumulation of excess mitochondrial Fe, *atfh* mutants are unable to direct their Fe reserves for proper utilization, resulting in compromised Fe-requiring biochemical reactions in the cell (Maliandi et al., 2011; Jain and Connolly, 2013). Interestingly, it has been suggested that frataxin functions in donating Fe for heme synthesis in plant mitochondria.(Armas et al., 2019). Thus, adequate supply of Fe and its proper utilization appears to be obligatory for embryogenesis and survival.

To validate their role in mitochondrial Fe transport *in planta*, we studied the effect of loss of MITs on mitochondrial Fe homeostasis. Loss of MIT1 and MIT2 results in reduced mitochondrial Fe but elevated mitochondrial Zn (**Figures 8** and **S2**). A few regulators have been reported in the literature which function to sense Fe availability based on the ratio of Fe to other metals such as Zn in the cell (Kobayashi et al., 2013). The Fe and Zn binding sites on these regulators sense the imbalance between the two elements to trigger the Fe deficiency response (Kobayashi et al., 2013). Although mitochondrial Fe sensors in plants are still unknown, the aforementioned mechanism may explain the up-regulation of the Fe deficiency pathway in response to the altered elemental profile of *mit1* ^−−^ */mit2* *^+^* ^−^ mitochondria (**Figure 8**). Reduced complex I activity and depleted aconitase levels in the mutants further substantiate the significance of MITs in mitochondrial iron trafficking and homeostasis (**Figures 8** and **S2**). These data corroborate the results of a recent study that used transcriptomic and metabolomic profiling to shed light upon the effects of *mit* knockdown on primary metabolism in rice (Vigani et al., 2016).

Mitochondrial iron transporters have been characterized in several species. While mitoferrins/MITs seem to be the major iron importers, they are not the sole transporters of Fe into the mitochondrial matrix. Mitoferrins/MITs appear to be particularly important during the early stages of development (**Figure 7**) (Muhlenhoff et al., 2003; Shaw et al., 2006; Bashir et al., 2011). In addition, the presence of other low affinity mitochondrial Fe transporters or non-specific divalent metal transporters have been reported in the literature (Yoon et al., 2011; Małas et al., 2018; Migocka et al., 2019); such transporters may be responsible for Fe import under normal to high cytosolic Fe conditions (Jain and Connolly 2013). Whether MIT1 and MIT2 can shuttle other ions other than Fe is not clear at this point but a role of their orthologs, Mfrn1, MRS3 and MRS4 in transporting other cations has been reported in the past (Muhlenhoff et al., 2003; Froschauer et al., 2009; Froschauer et al., 2009). In general, mitoferrins play a crucial role in heme and Fe-S cluster biosynthesis in yeast, zebrafish, and mammals; however, their role in plants may be limited to Fe-S cluster synthesis since definitive proof of mitochondrial heme synthesis in plants is lacking (Muhlenhoff et al., 2003; Shaw et al., 2006; Paradkar et al., 2009; Bashir et al., 2011; Jain and Connolly, 2013; Rouault, 2016). In fact, we measured total catalase activity (heme containing enzyme) and observed no difference in the activities in lysates prepared from WT and *mit* mutant backgrounds (data not shown). Nevertheless, MITs seem to be the major mitochondrial Fe transporters, and their significance in mitochondrial and cellular Fe homeostasis is clear.

Given that *mit* mutants displayed reduced Fe content and altered Fe metabolism in the mitochondria, we sought to examine Fe metabolism in whole tissues. The Arabidopsis *mit* mutants show an onset of the Fe deficiency response in roots (**Figure 10**), although shoot Fe content is not dramatically altered in *mit* lines, all the mutants show elevated accumulation of other divalent metal ions, such as Zn, Mn, and Co (**Figure 9**). This phenotype has been previously described as the Fe deficiency signature phenotype (Baxter et al., 2008; Walker and Connolly, 2008). In Arabidopsis, *IRT1* expression responds to Fe deficiency; however, IRT1 non-specifically transports various other metals, such as Mn, Co, Zn, and Cd, along with Fe (Connolly et al., 2002; Eide et al., 1996; Korshunova et al., 1999; Rogers et al., 2000; Vert et al., 2002). These results suggest that cells monitor mitochondrial Fe levels and upregulate the root Fe uptake machinery when mitochondrial Fe levels fall too low. In addition to this, *mit* mutants in Arabidopsis also exhibit significantly elevated shoot Cu levels. This is presumably because Fe deficiency is known to up-regulate a high affinity Copper Transporter (COPT2), which in turn leads to accumulation of Cu in the shoots (Perea-Garcia et al., 2013). This elevated Cu uptake is thought to aid in maintaining metal homeostasis as it allows the plant to switch from Fe-utilization to Cu-utilization pathways, which in turn, helps in the prioritization of Fe and eventual recovery from Fe deficiency (Yamasaki et al., 2009; Garcia et al., 2019).

The *MIT1* and *MIT2* promoters are active throughout the plant with the highest activity in the vasculature (**Figure 4**). The expression of both MIT1 and MIT2 has been observed as early as the stage of seed hydration, at a time when mitochondria become bioenergetically active (Paszkiewicz et al., 2017). However, the two genes are expressed at different levels at different stages of plant growth (www.travadb.org, Arabidopsis efpbrowser) which suggests that they may have different roles in iron metabolism. In recent years, significant progress has been made in elucidation of transcriptional networks that respond to Fe deficiency. One such network is the PYE network that functions in the pericycle (Long et al., 2010). According to this study, although MITs are expressed in the pericycle and the stele, their expression is not regulated by PYE or Fe availability (Dinneny et al., 2008; Long et al., 2010). Interestingly, while *MIT1* and *MIT2* are not highly regulated by Fe or Cu availability, *MIT1* is regulated by FIT (Mai et al., 2016) and *MIT1* and *MIT2* are regulated by SPL7 (the master regulator of copper-deficient responses) in roots and shoots, respectively (Bernal et al., 2012). This suggests that (similar to human mitoferrins) (Paradkar et al., 2009), while MIT1 and MIT2 are functionally redundant, they may have somewhat specialized functions that remain to be elucidated.

In summary, our results show that *Arabidopsis* MIT1 and MIT2 function in mitochondrial Fe import and together play an essential role in maintenance of cellular and mitochondrial Fe homeostasis. These proteins also are essential for embryogenesis and metabolism under iron-limiting conditions. It is interesting that Arabidopsis, a dicot, has two genes that encode mitochondrial Fe importers, while the monocot rice has just one. Furthermore, it is important to note that loss of MIT function in *Arabidopsis* as compared to rice affects Fe deficiency responses and elemental profiles differently; the consequences of these differences will be the basis for future studies. This work contributes to a comprehensive understanding of Fe homeostasis in plants which may, in turn, help in formulation of strategies to develop Fe-fortified food crops and/or crops that show enhanced performance on marginal soils.

## Data Availability Statement

All datasets generated for this study are included in the article/**Supplementary Material**.

## Author Contributions

AJ designed and performed the experiments, analyzed the data and wrote the manuscript. ZD performed qRT-PCR experiments on the mutant lines. EC designed and supervised the study, analyzed the data and wrote the manuscript. 

## Conflict of Interest

The authors declare that the research was conducted in the absence of any commercial or financial relationships that could be construed as a potential conflict of interest.
