# Supplementary material for: Mitochondrial Iron Transporters (MIT1 and MIT2) Are Essential for Iron Homeostasis and Embryogenesis in Arabidopsis thaliana
Source: Front Plant Sci. 2019 Nov 25;10:1449. doi: 10.3389/fpls.2019.01449 (PMC6889801; doi:10.3389/fpls.2019.01449)
Supplement: Supplementary file 1 [file Presentation_1.pdf]

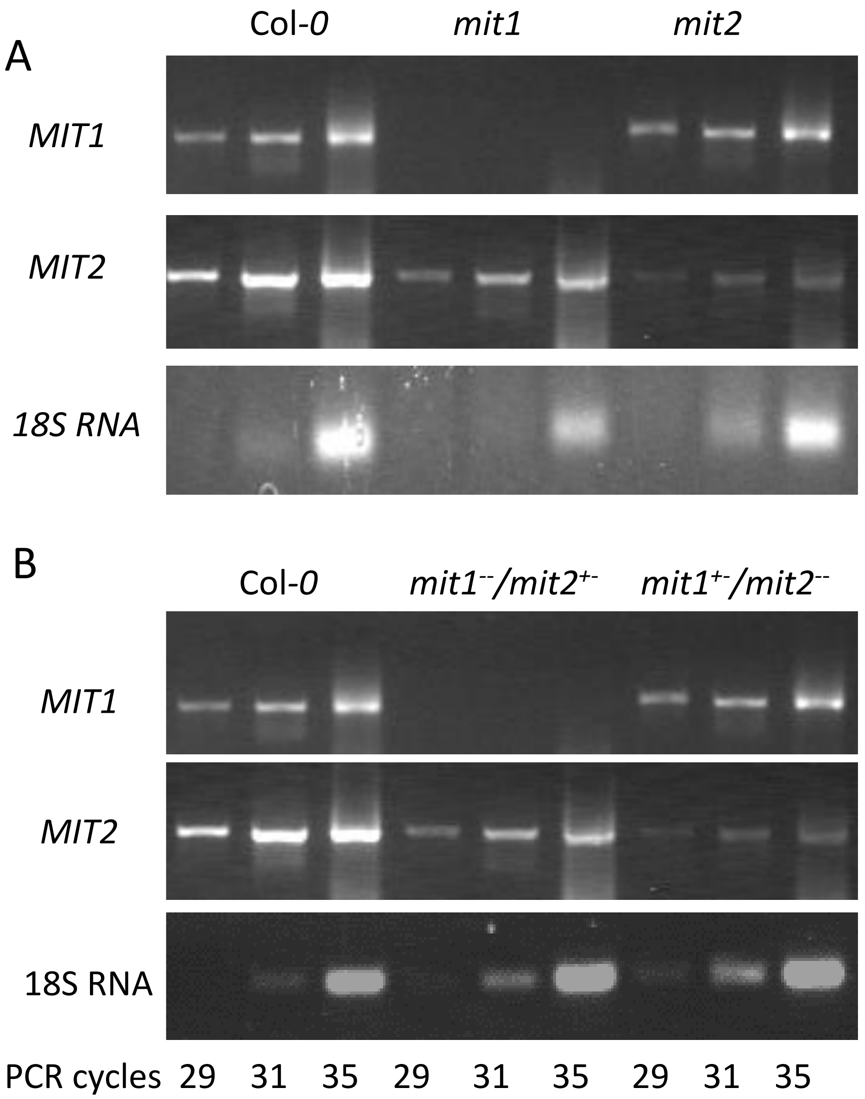

Figure S1: Genetic analysis of *mit1* and *mit2* mutants. (A) Analysis of *MIT1 and MIT2* gene expression by semi-qRT PCR in the single T-DNA insertion mutants (*mit1*, *mit2*) and (B) in the lines mutant for both *MIT1* and *MIT2* (*mit1*$^{--}$/*mit2*$^{+-}$, *mit1*$^{+-}$/*mit2*$^{--}$) grown in Fe-sufficient media for 2.5 weeks.

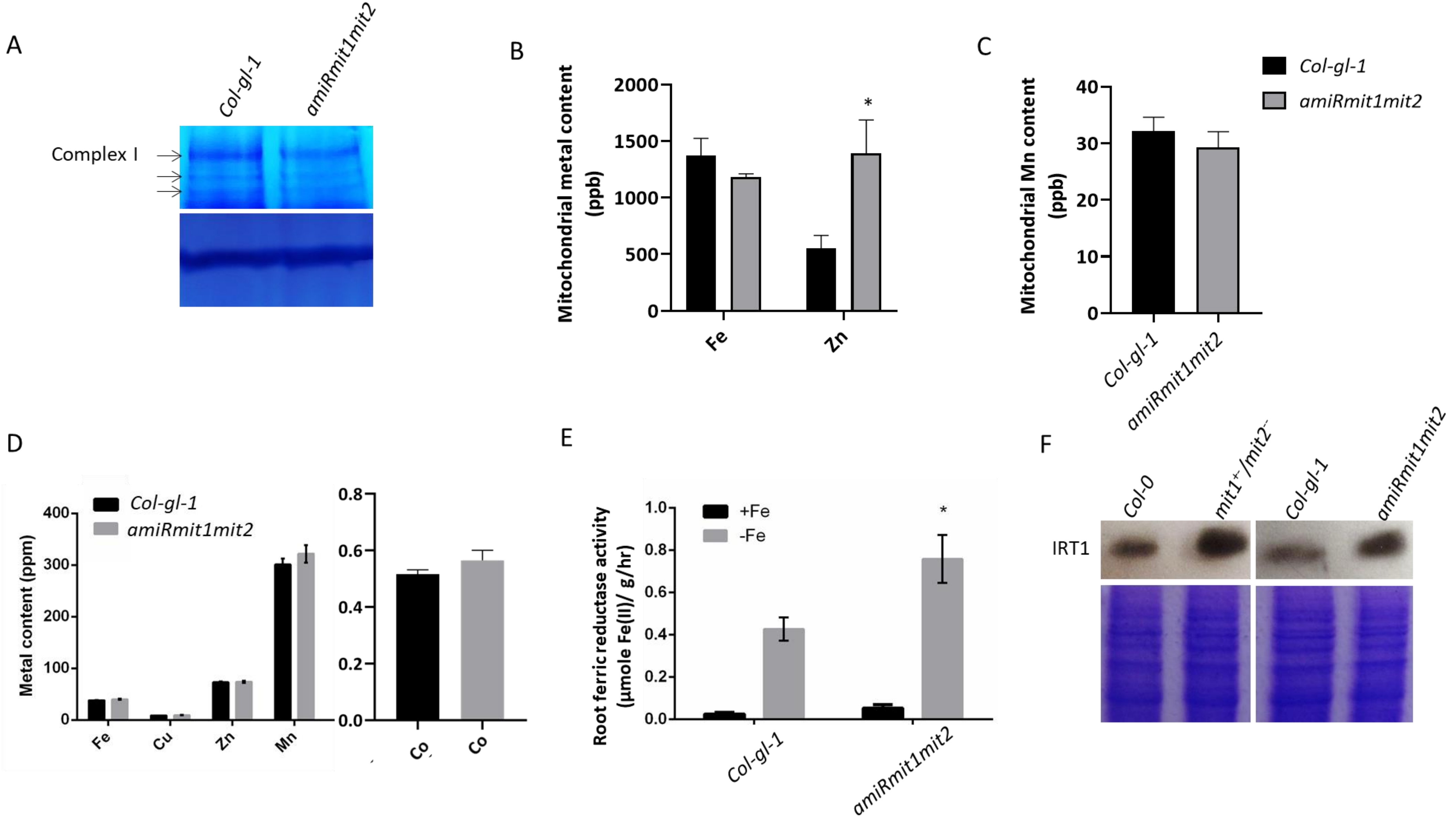

Figure S2: *amiRmit1mit2* mutant characterization. (A) In-gel enzyme activity staining for complex I on mitochondrial extract separated by BN-PAGE. The arrows indicate complex I and its supercomplexes. Non-specific staining at the bottom of the gel was used as the loading control. (B) Mitochondrial Fe, Zn and (C) Mn content measured by ICP-MS on plants grown in Fe-drop-out media. (D) ICP-MS metal profile of shoots of WT and *amiRmit1mit2* grown in soil. The data represents the average of 10 biological replicates. (E) Root ferric reductase activity assay on the seedlings grown in Fe sufficient or Fe deficient media. Values shown are an average of 10 biological replicates. (F) Immunoblot representing IRT1 levels in *mit* mutants grown in Fe deficient conditions. Coomassie stained internal proteins were used as loading control. Error bars in all the graphs indicate standard deviation and asterisk represents p value<0.05 (calculated by student's *t* test) compared to the WT.

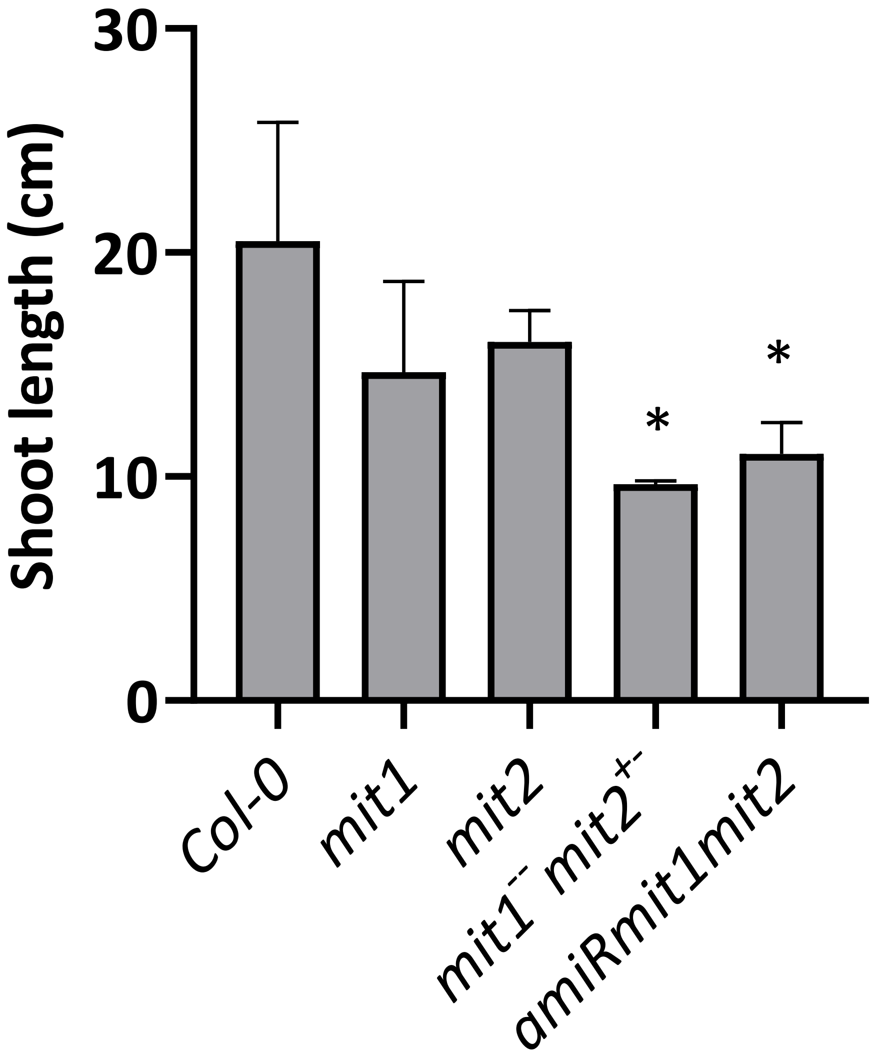

Figure S3: Comparison of shoot length of 5 week-old *mit* mutants grown hydroponically in Fe drop-out media. The data shows the average of 3-4 biological replicates. Significance ($p<0.05$) was assessed using the student's *t* test; significant differences in the shoot length as compared to WT is represented by an asterisk.
